# Inferring compound heterozygosity from large-scale exome sequencing data

**DOI:** 10.1101/2023.03.19.533370

**Published:** 2023-03-23

**Authors:** Michael H. Guo, Laurent C. Francioli, Sarah L. Stenton, Julia K. Goodrich, Nicholas A. Watts, Moriel Singer-Berk, Emily Groopman, Philip W. Darnowsky, Matthew Solomonson, Samantha Baxter, gnomAD Project Consortium, Grace Tiao, Benjamin M. Neale, Joel N. Hirschhorn, Heidi L. Rehm, Mark J. Daly, Anne O’Donnell-Luria, Konrad J. Karczewski, Daniel G. MacArthur, Kaitlin E. Samocha

**Affiliations:** 1 Department of Neurology, Hospital of the University of the Pennsylvania, Philadelphia, PA, USA; 2 Program in Medical and Population Genetics, Broad Institute of MIT and Harvard, Cambridge, MA, USA; 3 Analytic and Translational Genetics Unit, Massachusetts General Hospital, Boston, MA, USA; 4 Division of Genetics and Genomics, Boston Children’s Hospital, Boston, MA; 5 Stanley Center for Psychiatric Research, Broad Institute of MIT and Harvard, Cambridge, MA, USA; 6 Department of Genetics, Blavatnik Institute, Harvard Medical School, Boston, MA, USA; 7 Division of Endocrinology, Boston Children’s Hospital, Boston, MA, USA; 8 Center for Basic and Translational Obesity Research, Boston Children’s Hospital, Boston, MA, USA; 9 Center for Genomic Medicine, Massachusetts General Hospital, Boston, MA, USA; 10 Institute for Molecular Medicine Finland, (FIMM) Helsinki, Finland; 11 Centre for Population Genomics, Garvan Institute of Medical Research, UNSW Sydney, Sydney, Australia; 12 Centre for Population Genomics, Murdoch Children’s Research Institute, Melbourne, Australia

## Abstract

Severe recessive diseases arise when both the maternal and the paternal copies of a gene carry, or are impacted by, a damaging genetic variant in the affected individual. When a patient carries two different potentially causal variants, accurate diagnosis requires determining that these two variants occur on different copies of the chromosome (i.e., are in *trans*) rather than on the same copy (i.e., in *cis*). However, current approaches for determining phase, beyond parental testing, are limited in clinical settings. We developed a strategy for inferring phase for rare variant pairs within genes, leveraging haplotype patterns observed in exome sequencing data from the Genome Aggregation Database (gnomAD v2, n=125,748). When applied to trio data where phase is known, our approach estimates phase with high accuracy, even for very rare variants (frequency <1×10^−4^), and also correctly phases 95.2% of variant pairs in a set of 293 patients carrying presumed causal compound heterozygous variants. We provide a public resource of phasing estimates from gnomAD, including phasing estimates for coding variants across the genome and counts per gene of rare variants in *trans*, that can aid interpretation of rare co-occurring variants in the context of recessive disease.

## Introduction

Determination of haplotypic phase has important implications in clinical genetics, particularly in the diagnosis of recessive diseases that result from disruption of both copies of a gene. The disrupting bi-allelic variants can be either homozygous, where the same variant is present on both copies, or compound heterozygous, where two different variants are present on the two copies of the gene. Compound heterozygous variants present a challenge in genetic diagnosis because two variants observed within a gene in an individual can occur in *trans* or in *cis,* and only the former scenario results in compound heterozygosity. However, parental data are often not readily available for phasing (which is the typical situation in panel testing) or parents may not be available for follow-up testing, and short-read next generation sequencing largely cannot directly distinguish whether variant pairs are in *trans* or in *cis.* Thus, there is an important need for other approaches to accurately, easily, and cheaply determine phase of variant pairs.

The genetic relationship between a pair of variants on a haplotype can be disrupted by one of two processes: meiotic recombination and recurrent mutations. Meiotic recombination occurs more frequently in “hotspot” regions, and the probability of a recombination event occurring increases with distance between two variants^1^. A recurrent germline mutation event affecting a variant on a haplotype can also disrupt the genetic relationship of the variants on the haplotype. Rates of recurrent mutations are dependent on mutation type (e.g., transition versus transversion) and epigenetic marks (particularly CpG methylation), among other factors^2–6^. Thus, the rates of both meiotic recombination and mutation have important implications in determining the phase of variants.

There are several approaches for directly inferring phase for variant pairs observed in an individual. Phase may be determined directly using data from sequencing reads. However, for data from typical short-read sequencing technologies such as Illumina, read-based phasing methods are generally only possible for variants in close proximity to each other^7^, although some variant pairs at longer distances can be phased with more sophisticated algorithms^8–10^. Long-read sequencing technologies allow for direct determination of phase for variant pairs at longer genetic distances, but these technologies are more expensive and have not yet been widely applied in clinical settings^11,12^. There are also laboratory-based molecular methods for determining phase of variant pairs, but these methods are low-throughput and technically challenging^13^. While phase can be determined based on transmission of variants from parents to offspring, this approach increases cost and may pose other logistical and ethical challenges, and is not always an option if parents are deceased, unavailable (e.g., living far away or incarcerated), unknown in the case of adoption, or unwilling to participate. These direct phasing approaches all thus present critical limitations for determining phase of variant pairs within an individual in a clinical setting.

Alternative, indirect approaches to phasing rely on statistical methods applied to population data (reviewed in Tewhey et al.^14^ and Browning and Browning^15^). These approaches leverage genetic data from large numbers of unrelated or distantly related individuals, and rely on identifying shared haplotypes among individuals in a population. However, these methods require a large number of reference samples (typically n~10^5^-10^6^ individuals) and are computationally intensive to perform. These approaches perform less well for rare variants and cannot be applied to exome sequencing data, which does not provide enough density of surrounding variants to allow for accurate phasing. Despite these limitations, these population-based approaches are attractive because they do not require sequencing of additional family members or application of expensive sequencing approaches.

In this work, we sought to address existing challenges of phasing in clinical settings, particularly with regard to rare variants seen in exome sequencing data. We implement an approach that leverages the principles of population-based phasing by estimating haplotype patterns from a large reference population and using these patterns to infer variant phase in an individual. We cataloged the haplotype patterns of rare coding variants within genes using the Genome Aggregation Database (gnomAD), which performed aggregation and joint genotyping of exome sequencing data from 125,748 individuals^16^. We then demonstrate that we can leverage these data to generate a resource for phasing rare coding variants observed in an individual, and identify factors that influence the accuracy of our approach. Additionally, we provide statistics for how often different types of variants are observed, split by allele frequency and mutational consequence, in *trans* within gnomAD to provide a background rate contextualization when observing biallelic rare variants in rare disease cases. Finally, we disseminate these resources in a user-friendly fashion via the gnomAD browser for community use.

## Results

### Inference of phase in gnomAD

**Figure 1:**
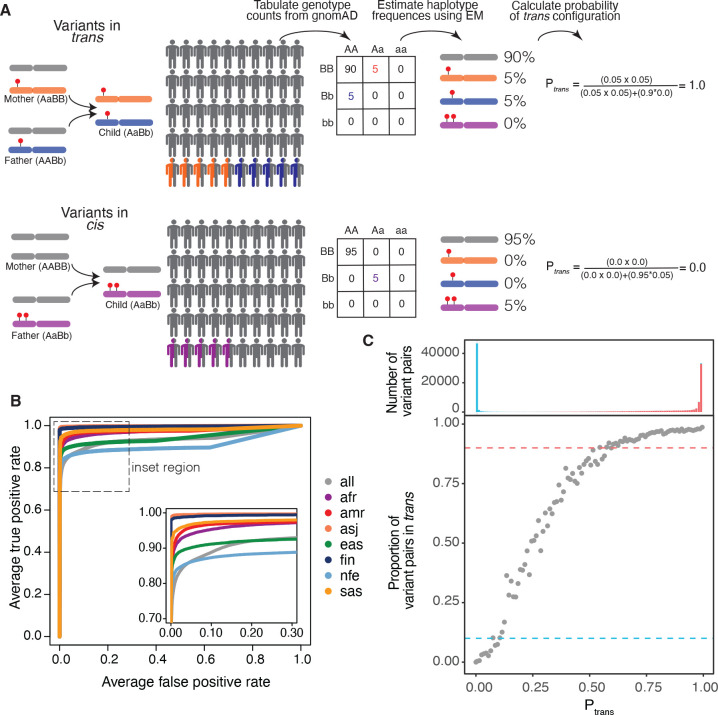
**A)** Schematic of phasing approach. **B)** Receiver-operator curves for use of $P_{\text{trans}}$ for distinguishing between variant pairs on same versus opposite haplotypes. Separate lines are shown for each genetic ancestry group. **C)** (bottom) Proportion of variant pairs in each $P_{\text{trans}}$ bin that are on opposite haplotypes. Each point represents variant pairs with $P_{\text{trans}}$ bin size of 0.01. Blue dashed line at 10% indicates the $P_{\text{trans}}$ threshold at which ≥90% of variant pairs in bin are on the same haplotype (Ptrans ≤0.10). Red dashed line at 90% indicates the $P_{\text{trans}}$ threshold at which ≥90% of variant pairs in bin are on opposite haplotypes ($P_{\text{trans}}\;\geq 0.55$). Calculations are performed using variant pairs where each variant in the pair has allele count > 100. $P_{\text{trans}}$ estimates are population-specific. (top) Histogram showing number of variant pairs in each $P_{\text{trans}}$ bin that are on the same haplotype (red) or opposite haplotypes (blue).

We sought to address the challenges of phasing individual samples in clinical settings by applying the principles of population-based phasing. Specifically, we leveraged the fact that haplotypes are usually shared across individuals in a population to infer phase of variants in an individual (Figure 1A). If two variants are in *cis* in many individuals in a population, then they are likely to be *cis* in any given individual’s DNA. Similarly, if two variants are in *trans* in other individuals in a population, then they are likely to be *trans* in any given individual’s DNA. This later scenario also provides information that the variant combination may be tolerated in *trans* since it has been found in an individual in gnomAD. We reasoned that by generating phasing estimates from a large reference population, we could infer the phase of variants observed in an individual.

Predicting the phase of a given pair of variants in an individual first requires that we estimate the haplotype frequencies in the population for a given pair of variants. To estimate haplotype frequencies, we used exome sequencing samples from gnomAD v2, a large sequencing aggregation database^16^. In total, there were 125,748 exome sequencing samples after rigorous sample and variant quality control (Methods). There are several key advantages of using gnomAD. First, samples in gnomAD undergo uniform processing and variant-calling, mitigating the impact of technical artifacts. Second, with over 250,000 chromosomes in gnomAD, the database provides sufficient sample sizes to estimate haplotype frequencies below 1×10^−5^. Lastly, gnomAD offers significant genetic ancestral diversity, allowing results of our study to be applied beyond samples with European genetic ancestry.

We focus in this study on pairs of rare coding variants occurring in the same gene, which are of the greatest interest in the context of Mendelian conditions. We required both variants to have a global minor allele frequency in gnomAD exomes <5% and required variants to be coding, flanking intronic (from position −1 to −3 in acceptor sites, and +1 to +8 in donor sites) or in the 5’/3’ UTRs. This encompassed 20,921,100 pairs of variants across 19,685 genes. We performed estimates based on all exome sequencing samples in gnomAD v2, as well as separate estimates within each of seven genetic ancestry groups, which ranged from 5,076 individuals of Ashkenazi Jewish genetic ancestry to 63,369 for non-Finnish European genetic ancestry.

For each pair of variants, we first generated pairwise genotype counts in gnomAD, with nine possible pairwise genotypes for each pair of variants (Figure 1A). We then applied the Expectation-Maximization (EM) algorithm to each pair of variants to generate haplotype frequency estimates based on the observed pairwise genotype counts^17^. For a given pair of variants observed in an individual, the probability of two variants being in *trans* ($P_{\text{trans}}$) is the probability of inheriting each of the haplotypes that contain only one of the two variant alleles.

### Validation of phasing estimates using trio data

To estimate the accuracy of our approach, we analyzed variants in a set of 4,992 trios that were exome sequenced and jointly processed with gnomAD, but which are independent of the samples included in the gnomAD release. The trio structure allowed us to accurately determine phase by transmission in these samples. We estimated the genetic ancestry of each individual in the trios by projecting on the principal components of ancestry in the gnomAD v2 samples (Figure S1). In order to maximize the number of variant pairs for testing, we utilized the variant pairs observed in either parent and used the child’s genotype to phase the parental genotypes. Separately, we used our population-based method leveraging gnomAD data to estimate phase for every pair of rare coding variants observed in either of the parents. Across the 4,992 trios, we identified 434,326 pairs of variants where both variants were observed at least once in gnomAD and thus were amenable to phasing using our approach. 62,363 pairs of variants were seen in multiple individuals, resulting in 371,963 unique pairs of variants. As described above, we calculated the probability of being in *trans* ($P_{\text{trans}}$) based on the haplotype frequency estimates in gnomAD. We found a bimodal distribution of $P_{\text{trans}}$ scores; that is, the majority of probabilities were either very high (>0.99; suggesting a high likelihood of being in *trans*), or they were very low (<0.01; suggesting a high likelihood of being in *cis*) (Figure S2).

Using the trio phasing-by-transmission data as a gold standard, we generated receiver-operator curves for distinguishing whether a variant pair is likely in *trans* and found that our approach achieved high sensitivity and specificity (AUC ranging from 0.905 to 0.998 across the component genetic ancestry groups) (Figure 1B) and high precision and recall (Figure S3).

We next defined $P_{\text{trans}}$ thresholds for classifying variants as being in *cis* versus *trans* (see Methods for additional details). To set these thresholds, we first binned variant pairs observed in the trio data based on their $P_{\text{trans}}$. For each bin, we calculated the proportion of trio variant pairs that were in *cis* or *trans* based on trio phasing-by-transmission. The $P_{\text{trans}}$ threshold for variant pairs in *trans* was defined as the minimum $P_{\text{trans}}$ such that ≥90% of variant pairs in that bin were in *trans* based on trio phasing-by-transmission. Similarly, the $P_{\text{trans}}$ threshold for variants in *cis* was defined as the maximum $P_{\text{trans}}$ such that ≥90% of variant pairs in that bin were in *cis* based on trio phasing by transmission. This resulted in $P_{\text{trans}}$ thresholds of ≤0.10 and ≥0.55 as the threshold for variants in *cis* and *trans*, respectively (Figure 1C).

Based on these $P_{\text{trans}}$ thresholds, the overall phasing accuracy was 93.5%. The accuracy for variants that are in *cis* based on trio data was 92.1%, and the accuracy for variants in *trans* was 94.7%. Only 6.72% of variants had indeterminate $P_{\text{trans}}$ ($0.10<M_{ss}<0.55$). Together, these results suggest that our approach can generate accurate phasing estimates.

### Accuracy of phasing across allele frequencies

**Figure 2:**
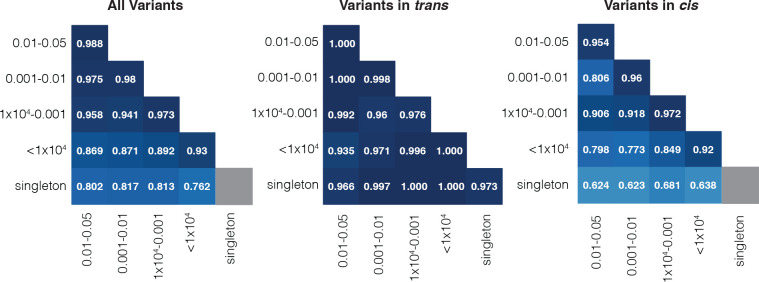
Phasing accuracy at different allele frequency bins for all variants (left), variants on opposite haplotypes (middle), and same haplotype (right). Shading of squares and numbers in each square represent the accuracy. Y-axis labels refer to the more frequent variant in each variant pair. X-axis labels refer to the rarer variant in each variant pair. Here, accuracy is the proportion of correct classifications (i.e., correct classifications / all classifications). Accuracies are calculated for variants seen in non-Finnish European samples based on population-specific $P_{\text{trans}}$ calculations.

Since the variants that are most likely to be of interest in clinical genetics are rare, we assessed the accuracy of phasing at different allele frequency (AF) bins. Using $P_{\text{trans}}$ estimates derived from non-Finnish European samples, we found high accuracy (i.e., proportion correct classifications) ranging from 0.76 to 0.99 across pairs of AF bins (Figure 2). In general, accuracy remained high across allele frequencies for variant pairs in *trans* based on trio phasing data. For variant pairs in *cis* based on trio phasing data, accuracy was high when variant pairs were of similar allele frequencies. However, accuracy was much lower, as low as 0.62, for variant pairs where one variant was much more common than the other. Variant pairs where both variants are singletons were phased well for variants in *trans* based on the trio phasing data (0.97). Given the lack of information, we do not report the phasing estimates for singleton/singleton variant pairs in *cis* (see Discussion).

### Accuracy of phasing across genetic ancestry groups

**Figure 3:**
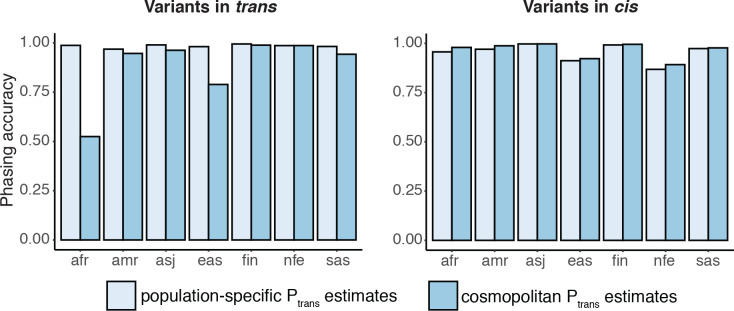
Phasing accuracy using population-specific $P_{\text{trans}}$ estimates (light blue) or cosmopolitan $P_{\text{trans}}$ estimates (medium blue). Accuracies are shown separately for variants in *trans* (left) and variants in *cis* (right)

In the above analyses, we used $P_{\text{trans}}$ estimates calculated from the genetic ancestry in which the variant was seen in the trio data. We next asked if using all samples in gnomAD to calculate $P_{\text{trans}}$ (“cosmopolitan”) would improve accuracy given larger sample sizes from which to calculate $P_{\text{trans}}$, with the caveat that using the full set of gnomAD samples would result in some genetic ancestry mismatching. Across genetic ancestry groups, we found that accuracy was similar when using population-specific ancestry estimates as compared to cosmopolitan estimates (Figure 3). However, for certain genetic ancestry groups such as African (AFR) and East Asian (EAS), accuracy was notably lower when using cosmopolitan estimates as compared to population-specific estimates specifically for variants in *trans* in these populations; for example, the phasing accuracy for variants in trans in the AFR ancestry group is close to 1 when using AFR-specific $P_{\text{trans}}$ estimates, but drops to ~0.5 when cosmopolitan $P_{\text{trans}}$ estimates are used. These results suggest that cosmopolitan estimates generally achieve similar accuracy as population-specific estimates, though we do identify certain scenarios where more caution is required.

### Effect of variant distance and mutation rates on phasing accuracy

**Figure 4:**
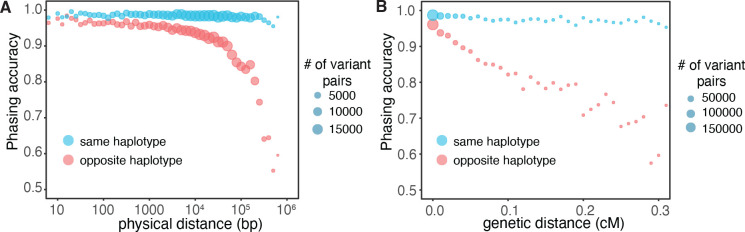
**A)** Phasing accuracy (y-axis) as a function of physical distance (in base pairs on log_10_ scale) between variants (x-axis). Red represents variants on the same haplotype, and blue represents variants on opposite haplotypes. **B)** Same as **A,** except the x-axis shows genetic distance (in centiMorgans). Accuracies for **A)** and **B)** are based on variant pairs seen in all populations and utilize population-specific $P_{\text{trans}}$ estimates.

One factor that is predicted to affect the accuracy of our phasing estimates is the frequency of meiotic recombination between pairs of variants, as recombination events would disrupt the haplotype configuration of the variant pairs. To further explore the impact of recombination, we plotted the accuracy of our phasing estimates as a function of physical distance between variant pairs. We found that for variants in *trans,* the accuracy of phasing was maintained across physical distances. However, for variant pairs in *cis,* the accuracy rapidly decreased with longer physical distances (Figure 4A). Nonetheless, within the scale of typical gene lengths (less than 100,000 base pairs), phasing accuracy was relatively preserved.

Since physical distance is only a proxy for recombination frequency, we also performed this analysis using interpolated genetic distances (Figure 4B). We found again that variants in *trans* had preserved phasing accuracy across genetic distances, while variants in *cis* had phasing estimates that decreased substantially with genetic distance, particularly at distances greater than 0.1 centiMorgan.

We also tested the effect of recombination by examining a set of 20,319 multinucleotide variants (MNVs), which are pairs of genetic variants in *cis* that are very close together in physical distance (≤2 bp) and thus have minimal opportunity for recombination between them. These variants have previously been accurately phased using physical read data^18,19^. When examining this set of MNVs, we found that the phasing accuracy using our approach was 98.9%, with only 0.60% that were phased incorrectly (the remaining 0.45% had indeterminate phasing estimates). We found that the vast majority of MNVs that were phased incorrectly using $P_{\text{trans}}$ had disparate AFs between the two variants, similar to what we had observed above (data not shown).

Another potential driver of inaccurate phasing is recurrent germline mutations, which depend in part on the type of mutation. Transitions have higher mutation rates than transversions^2,20^. Furthermore, CpG transitions have the highest mutation rates among single nucleotide changes, with mutation rates increasing with higher methylation rates at the CpG sites^16^. To better understand the impact of mutation rates, we classified each single nucleotide variant (SNV) in the trio data as a transversion, non-CpG transition, or CpG transition. For CpG transitions, we further classified the SNV as having low, medium, or high DNA methylation as before^16^. We then calculated phasing accuracy as a function of combinations of mutation types using the trio data (Figure S4). We found similar accuracy for transversions and transitions (~0.96). However, we found that CpG sites had lower phasing accuracy, particularly for variant pairs in *cis.* The phasing accuracies were lower at medium and high methylation CpG sites (0.88–0.94) than they were for low methylation sites (0.93–0.97). These results are consistent with recurrent mutations contributing to inaccurate phasing estimates.

### Demonstration in a cohort of patients with Mendelian disorders

To demonstrate our approach in a clinically relevant situation, we turned to a set of 627 patients from the Broad Institute Center for Mendelian Genetics (CMG)^21^. All patients had a confident or strong candidate genetic diagnosis of a Mendelian condition based on carrying two rare variants in a recessive disease gene consistent with the patient’s phenotype. Across the 627 diagnoses, we were able to estimate phase for the 293 patients in which both variants were present in gnomAD (Supplemental Table 1). Of these 293 variant pairs, our phasing method indicated 279 (95.2%) to be in *trans*, nine (3.1%) to be in *cis*, and five (1.7%) were indeterminate ($P_{\text{trans}}$ >0.10 and <0.55 or singleton-singleton variant in the same individual). Of the nine variant pairs indicated to be in *cis*, six originated from patients with proband-only sequencing. For these patients, the responsible clinician was contacted to ensure phenotype overlap with the disease gene and to pursue parental Sanger sequencing for confirmatory phasing by transmission, where possible. The remaining three variant pairs indicated to be in *cis* originated from patients with trio-sequencing, which confirmed the variants to be in *trans*, and our inferred phase to be incorrect. Despite a small number of errors, overall, the results suggest that our phasing approach is highly accurate in clinical scenarios in patients with suspected Mendelian conditions and can be informative for a large fraction (just under 50% in our cohort) of candidate diagnoses, dependant on presence of both variants in gnomAD.

### Bi-allelic predicted damaging variants

Next, we tabulated for each gene the number of individuals in gnomAD who carry two rare heterozygous variants, stratified by the predicted phase (e.g., in *trans,* unphased, and in *cis*), allele frequency, and the predicted functional consequence of the least damaging variant in the pair. For comparison, we tabulated individuals with homozygous variants in the same manner. We classified predicted functional consequences as predicted loss-of-function (pLoF), missense with deleteriousness scored by REVEL^22^ in line with recent ClinGen recommendations^23^, and synonymous. These data are available for gnomAD v2 as a downloadable table with the counts of individuals by phase for each gene across a total of 26 consequences and five allele frequency thresholds.

**Figure 5:**
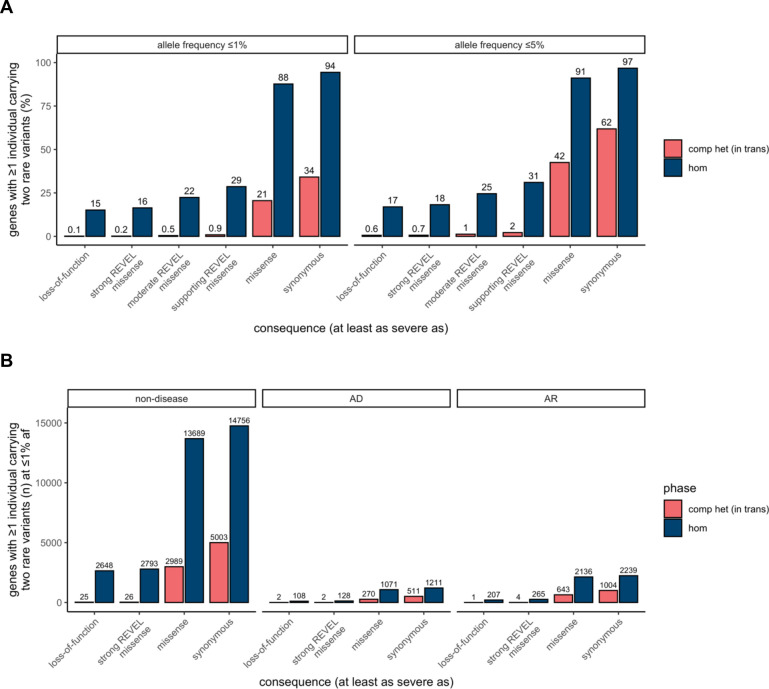
**A)** Proportion of genes with one or more individuals in gnomAD carrying predicted compound heterozygous (in *trans*) variants or a homozygous variant at ≤1% and ≤5% allele frequency stratified by predicted functional consequence. **B)** Number of genes with ≥1 individual in gnomAD carrying compound heterozygous (in *trans*) or homozygous predicted damaging variants at ≤1% allele frequency, stratified by predicted functional consequence and Mendelian disease-association in the Online Mendelian Inheritance in Man database (OMIM). In total, 28 genes (25 non-disease, 2 AD, and 1 AR) carried predicted compound heterozygous loss-of-function variants at ≤1% allele frequency, only seven of which were high confidence “human knock-out” events following manual curation. For predicted compound heterozygous variants, both variants in the variant pair must be annotated with a consequence at least as severe as the consequence listed (i.e., a compound heterozygous loss-of-function variant would be counted under the LoF category but also included with a less deleterious variant under the other categories). All homozygous pLoF variants previously underwent manual curation as part of Karczewski et al^16^. AF, allele frequency; comp het, compound heterozygous; hom, homozygous; AD, autosomal dominant; AR, autosomal recessive.

Overall, the number of individuals with rare, compound heterozygous (in *trans*), predicted damaging variants was low (median 0 individuals per gene with compound heterozygous loss-of-function variants at 1% allele frequency, range 0–9) and only occurred in a small number of genes (Figure 5 and Figure S5). Twenty eight genes carried compound heterozygous pLoF variants (in 56 individuals) and an additional four genes carried compound heterozygous variants with at least a strong REVEL missense predicted consequence (in six individuals) at 1% allele frequency. The vast majority of these genes have not, to date, been associated with disease (Figure 5B). Manual curation of the pLoF variants resulted in seven high confidence “human knock-out” genes (*ARHGEF37, CCDC66, FAM81B, FYB2, GNLY, RBKS,* and *SDSL*). These genes are not associated with Mendelian disease nor are they known to be essential (see Methods for additional details). In the remaining 21 of the 28 genes with compound heterozygous pLoF variants, true loss-of-function was found to be uncertain or unlikely following manual curation, due, for example, to the variant falling in the last exon of the gene, in a weakly conserved exon, or in the minority of transcripts (Supplemental Table 2). We previously manually curated all homozygous pLoF variants in gnomAD^16^. The absence of rare compound heterozygous “human knock-out” events in essential genes was expected given that gnomAD is largely depleted of individuals with severe, early-onset conditions.

### Generation of public resource

**Figure 6:**
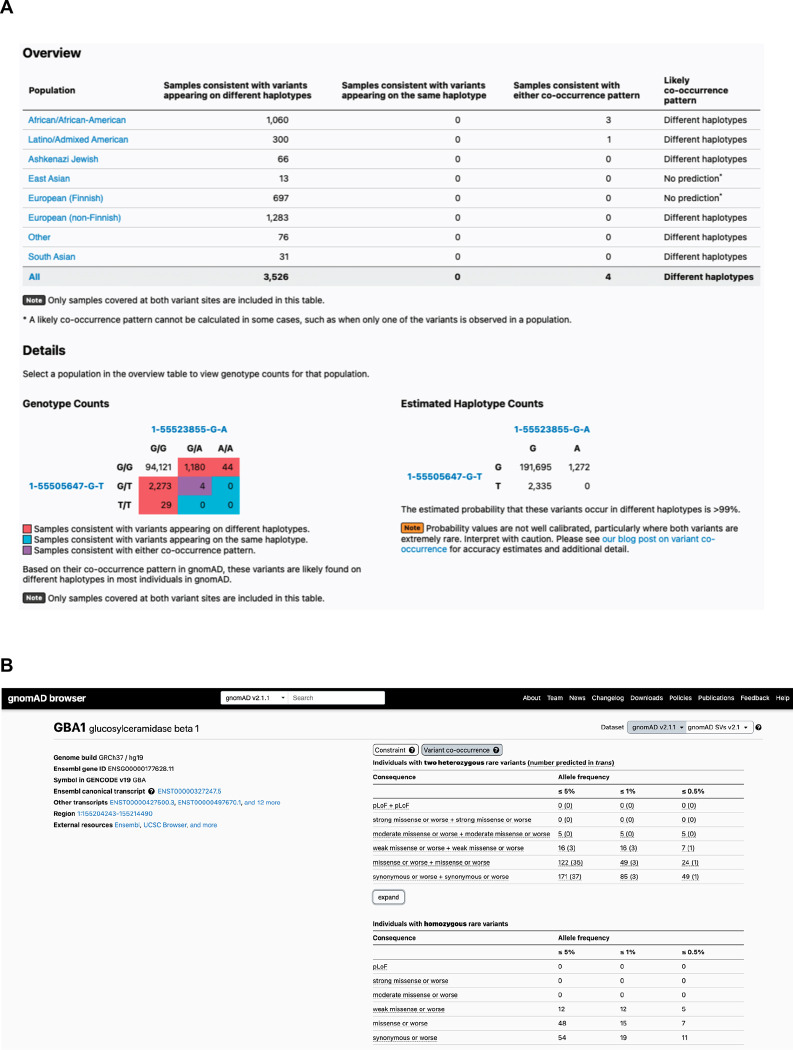
**A)** Sample gnomAD browser output for two variants (1-55505647-G-T and 1-55523855-G-A) in the gene *PCSK9*. On the top, a table subdivided by genetic ancestry group displays how many individuals in gnomAD from that genetic ancestry are consistent with the two variants occurring on different haplotypes (*trans*), and how many individuals are consistent with their occurring on the same haplotype (*cis*). Below that, there is a 3×3 table that contains the 9 possible combinations of genotypes for the two variants of interest. The number of individuals in gnomAD that fall in each of these combinations are shown and are colored by whether they are consistent with variants falling on different haplotypes (red) or the same haplotype (blue), or whether they are indeterminate (purple). The estimated haplotype counts for the four possible haplotypes for the two variants as calculated by the EM algorithm is displayed on the bottom right. The probability of being in *trans* for this particular pair of variants is > 99%. **B)** Variant co-occurrence tables on the gene landing page. For each gene (*GBA1* shown), the top table lists the number of individuals carrying pairs of rare heterozygous variants by inferred phase, allele frequency, and predicted functional consequence. The number of individuals with homozygous variants are tabulated in the same manner and presented as a comparison below. Allele frequency thresholds of ≤ 5%, ≤ 1%, and ≤ 0.5% are displayed across six predicted functional consequences (combinations of pLoF, various evidence strengths of predicted pathogenicity for missense variants, and synonymous variants). Both variants in the variant pair must be annotated with a consequence at least as severe as the consequence listed (i.e., pLoF + strong missense also includes pLoF + pLoF).

To make our resource widely usable to both clinicians and researchers, we have calculated and released pairwise genotype counts and phasing estimates for each pair of rare coding variants occurring in the same gene for gnomAD. These genotype counts are phasing estimates shown for all pairs of variants within a gene where both variants have global minor allele frequency (or population-specific frequency) in gnomAD exomes <5%, and are either coding, flanking intronic (from position −1 to −3 in acceptor sites, and +1 to +8 in donor sites) or in the 5’/3’ UTRs. We have integrated these data into the gnomAD browser so that clinicians and researchers can easily look up a pair of variants to obtain the genotype counts, haplotype frequency estimates, $P_{\text{trans}}$ estimates, and likely co-occurrence pattern (Figure 6A). These results are shown for each individual genetic ancestry group and across all genetic ancestries in gnomAD v2. In addition, the data are available as a downloadable table for all variants.

Furthermore, on the landing page of each gene in the gnomAD v2 browser, we have incorporated counts tables detailing the number of individuals carrying two rare variants stratified by allele frequency, and functional consequence. The first table counts individuals carrying two rare heterozygous variants by predicted phase (in *trans*, unphased, and in *cis*) and the second table counts individuals carrying homozygous variants (Figure 6B). We envision that these data will aid the medical genetics community in interpreting the clinical significance of co-occurring variants in the context of recessive conditions. The data for all genes are also available as a downloadable table within gnomAD v2.

## Discussion

In this work, we have leveraged a large exome sequencing cohort to estimate haplotype frequencies for pairs of rare variants within genes, and demonstrate that these haplotype frequency estimates can be utilized to predict phase of pairs of variants. Overall, we achieve high accuracy across a range of allele frequencies and across genetic ancestries and demonstrate that our approach is able to distinguish variants that are likely compound heterozygous in a clinical setting. Finally, we freely disseminate our results in an easy-to-use browser for the community.

Our phasing approach was accurate across a range of allele frequencies (even for singleton variants) and across genetic ancestry groups. However, we identified meiotic recombination as one key driver of decreased phasing accuracy. We found that for variants in *cis,* phasing accuracy diminished linearly with genetic distance, consistent with the role of meiotic recombination in decreasing phasing accuracy. However, for variants in *trans,* phasing accuracy was maintained across genetic and physical distances between pairs of variants. This result is intuitive, as for rare variants, a recombination event is much more likely to disrupt a haplotype comprised of two variants than to bring two rare variants onto the same haplotype. These results suggest the need for caution when using these phasing estimates for variants at long distances from each other where the phasing prediction is *cis.*

We also compared population-specific estimates with phasing estimates derived from samples across all genetic ancestry groups in gnomAD v2 (“cosmopolitan”). While population-specific phasing estimates are more likely to match the haplotypes seen in a given individual, they utilize information from fewer samples in gnomAD. We found that, in general, population-specific estimates were similar in accuracy to using cosmopolitan estimates. For individuals of African genetic ancestry, however, we found that use of cosmopolitan estimates resulted in much lower phasing accuracy than the use of African-specific estimates. This is consistent with the observation that there are more unique haplotypes seen in individuals of African genetic ancestry and/or older haplotypes in individuals of African genetic ancestry for which recombination is more likely to have occurred^24^. Moreover, there are other genetic ancestry groups not currently represented in gnomAD for which we expect this phasing approach to have lower accuracy than in the well-represented populations.

Pairs of singleton variants pose a unique challenge. When a pair of singleton variants is observed in different individuals in a population, this provides evidence that the variants are on different haplotypes. However, if a pair of singleton variants is observed in the same individual in the population, we cannot readily distinguish whether the variants are on the same haplotype or different haplotypes as we lack information from other individuals in the population for singleton variants. For this reason, we have chosen to not report phasing estimates for singleton variant pairs that are observed in the same individuals in gnomAD. Nonetheless, using our trio data, 93% of these singleton variant pairs observed in the same individual in gnomAD were in *cis* based on our trio validation data.

Our work focuses on the challenging scenario of determining phase for rare variants identified during sequencing of rare disease patients in the absence of parental data. Exome and targeted gene panel sequencing poses a unique challenge for population-based phasing given the sparsity of variants, precluding the use of common non-coding variants as a “scaffold” for phasing. One recent work performed population-based phasing of rare variants from exome sequencing data by combining the exome data with SNP genotyping arrays^25^. However, SNP genotyping data are not usually generated in conjunction with a sequencing test. Given this limitation of population-based phasing of exome sequencing data, we chose to apply the EM algorithm on pairs of rare variants. Rare variants, which are of the greatest interest in Mendelian diseases, are also challenging to phase using population-based approaches given the small numbers of shared haplotypes from which to make phasing estimates in the population. Recent methods have shown accurate phasing of rare variants using genome sequencing data^25–27^, but relies on a large genome reference panel. In our work, there were limited numbers of genome sequences available for use in a population-based phasing approach. However, as the numbers of genome sequencing samples increases in future releases of gnomAD, this may represent a tractable and more accurate approach for phasing of rare variants.

Utilizing phase estimates to tabulate the number of individuals in gnomAD with two rare predicted in *trans* variants by gene, we found that there are only a small number of “human knock-out” genes affected by predicted compound heterozygous (in *trans*) loss-of-function variants, and that this number is substantially lower than is observed for homozygous loss-of-function variants. These compound heterozygous “human knock-out” events occurred in genes that are not known to be essential, an expected finding given that gnomAD is largely depleted of individuals with severe and early-onset conditions. When analyzing the 23,667 individuals that carry two pLoF variants with allele frequency ≤ 1%, we predict that in 20,706 (88%) of those individuals, the variant pair is in *cis* and only a small fraction (~0.2%) are confidently predicted to be in *trans*. This may be counter-intuitive, so warrants emphasis: when a pair of rare pLoF variants is observed in the same gene in an individual from a general population sample, it is vastly more likely that these variants are carried on the same haplotype than that the individual is a genuine “knock-out” due to compound heterozygosity.

To aid the medical genetics community in interpreting the clinical significance of rare co-occurring variants in the context of recessive disease, we have released these counts across a spectrum of variant consequences (loss-of-function, missense, and synonymous) and allele frequencies by gene in the gnomAD browser. These provide background frequencies–to the best of our ability–of compound heterozygous rare damaging variants. These background frequencies can be used to assess the probability that a given variant pair identified in a patient may have occurred by chance. We note, however, that our ability to identify rare variant pairs in *trans* in gnomAD v2 is limited by the fact this dataset was used for training. We find that our ability to detect in *trans* variant pairs extends only to an allele frequency of 0.5%, with all rarer combinations being dominated by unphased predictions and very few predictions of *in trans* variant pairs. The per-gene variant co-occurrence resource developed and released here is therefore to be considered a first step in this space. We plan to use the predictions from this dataset (gnomAD v2) on newer versions of gnomAD with additional samples, where we can confidently predict rare variant pairs that are in *trans*.

Beyond our current restriction to predicting within the gnomAD dataset, there are several other important limitations to our work. First, we have only reported phasing estimates for rare coding and intronic flanking/UTR variant pairs within genes in order to limit the computational burden. We believe that these are the variant pairs of most interest to the medical genetics community, but acknowledge that phase with deeper intronic variation will become important as more genome sequencing is performed. Second, while there was a broad range of genetic ancestral diversity represented in our samples, future studies would benefit from even larger sample sizes, especially for genetic ancestry groups not well represented in our present study. Finally, we have only tested our phasing accuracy in a clinical setting in a retrospective manner and future prospective studies will be needed to confirm the clinical utility of our approach.

## Methods

### gnomAD characteristics and data processing

In this work, we used exome sequencing data from the gnomAD v2.1 dataset (n = 125,748 individuals). These data were uniformly processed, underwent joint variant calling, and rigorous quality control, as described in Karczewski et al.^16^. Briefly, we aggregated ~200k exome sequences and ~20k genome sequences, primarily from case-control studies of common adult-onset conditions, and applied a BWA-Picard-GATK pipeline^28^. Using Hail (https://github.com/hail-is/hail), we then removed samples that (1) failed population- and platform-specific quality control, (2) had second-degree or closer relations in the dataset, (3) did not have appropriate consent for release, and (4) had known severe, early-onset conditions. For variant quality control, we trained a random forest on site-level and genotype-level metrics (e.g., the quality by depth, QD), and demonstrated that it achieved both high precision and recall for both common and rare variants.

We subsetted the final cleaned gnomAD dataset for variants with global allele frequency in gnomAD exomes <5% that were either coding, flanking intronic (from position −1 to −3 in acceptor sites, and +1 to +8 in donor sites) or in the 5’/3’ UTRs. This encompassed 20,921,100 pairs of variants across 19,685 genes.

Analysis in this manuscript was performed using Hail version 0.2.105^29^, and analysis code is available at https://github.com/broadinstitute/gnomad_chets.

### Haplotype estimates

Consider two variants, A and B. A and B represent the major alleles, and a and b represent the respective minor alleles. There are thus 9 pairwise genotypes for A and B: AABB, AaBB, aaBB, AABb, AaBb, aaBb, AAbb, Aabb, and aabb. Of these pairwise genotypes, only the phase for the double heterozygote (AaBb) is unknown. From these 9 possible genotypes, there are four possible haplotype configurations: AB, Ab, aB, and ab.

For each pair of variants, we applied the expectation-maximization (EM) algorithm^17^ to estimate haplotype frequencies from genotype counts. The initial conditions of the EM algorithm were set by partitioning the doubly heterozygous (AaBb) genotype counts equally between the AB|ab and Ab|aB haplotype configurations. The EM algorithm was run until convergence or until the absolute value of the difference between consecutive maximum likelihood function values was less than 1×10^−7^. We calculated haplotype frequencies based on genotypes present within the same population (“population-specific”) or using all samples from gnomAD (“cosmopolitan”). Haplotype frequency estimates were performed using Hail.

We then calculate $P_{\text{trans}}$ as the likelihood that any given pair of doubly heterozygous variants (AaBb) in a patient is compound heterozygous (Ab|aB). $P_{\text{trans}}$ can be calculated simply from the haplotype frequency estimates (AB,Ab,aB, and ab):

$$P_{\text{trans}\;}=((Ab\times aB))/(AB\times ab+Ab\times aB)$$

Thus, $P_{\text{trans}}$ simply represents the probability that the patient is compound heterozygous by inheriting both the Ab and aB haplotypes.

### Determination of $P_{\text{trans}}$ cutoffs

To determine $P_{\text{trans}}$ cutoffs for classifying variants as occurring in *cis* or *trans*, we binned variant pairs in increments of 0.01 of $P_{\text{trans}}$. For each bin, we calculated the proportion of variant pairs in that bin that are compound heterozygous based on phasing by trio data. The $P_{\text{trans}}$ for compound heterozygous variants was determined as the minimum $P_{\text{trans}}$ such that 90% of variants in the bin are compound heterozygous based on trio data. The $P_{\text{trans}}$ for variants in *cis* was determined as the maximum $P_{\text{trans}}$ such that 90% of variants in the bin are in *cis* based on trio data. For these calculations, we used only variants where both variants had a population allele count greater than 100. We used population-specific $P_{\text{trans}}$ estimates.

### Trio validation data

Part of the original joint calling of gnomAD included exome sequencing data from full parent-child trios. Since most of these trios came from cohorts where the child had a severe, early-onset condition, the trios were removed from the final release of gnomAD but were similarly processed as the released samples. Here, we made use of 4,992 trios that were processed with gnomAD but not released. Having access to parental information allows us to perform phase-by-transmission and accurately determine whether two co-occurring variants in the same gene are in *cis* or in *trans*.

First, we estimated genetic ancestry of each individual in the trios by using ancestry inference estimates from the full gnomAD dataset, as previously described^16^. Briefly, we selected bi-allelic variants that passed all hard filters, had allele frequencies in a joint exome and genome callset > 0.001, and high joint call rates (> 0.99). The variants were then LD-pruned (r^2^ = 0.1) and used in a principal component analysis (PCA). We previously used samples with known genetic ancestry to train a random forest on the first 20 principal components (PCs), and assigned samples to a genetic ancestry group based on having a random forest probability > 0.9. For the trios in this cohort, we projected their PCs for genetic ancestry onto the same gnomAD v2 samples to infer the population used here (Figure S1).

We then phased the trio data using the Hail *phase_by_transmission* (https://hail.is/docs/0.2/experimental/index.html#hail.experimental.phase_by_transmission) function, which uses Mendelian transmission of alleles to infer haplotypes in trios for all sites that aren’t heterozygous in all members of the trio. Assigning haplotypes in trios based on parental genotype has traditionally been the gold standard, has switch error rates below 0.1%, and importantly errors aren’t dependent on the allele frequency of the variants phased^30^. To maximize our confidence in the genotypes and phasing, we filtered genotypes to include only those with genotype quality (GQ) > 20, depth > 10 and allele balance > 0.2 for heterozygous genotypes prior to phasing. Sex chromosomes were excluded. Using these criteria, we ended up with 434,326 variant pairs where both of the variants were observed at least once in gnomAD and could thus be used to evaluate accuracy of our method.

### CpG analysis

Single nucleotide variants seen in the trio data were divided into transitions and transversions. Transitions were further subdivided into those that are CpG mutations (5’-CpG-3’ mutating to 5’-TpG-3’) and those that are not. For each CpG transition, we calculated the mean DNA methylation values across 37 tissues in ENCODE^16^. We then stratified CpG transitions into 3 levels: low (missing or < 0.2), medium (0.2–0.6), and high (>0.6) methylation. Phasing accuracy–here, the proportion correct (correct classifications/all classifications)–was then calculated for pairwise combinations of transversions, non-CpG transitions, low methylation CpG transitions, medium methylation CpG transitions, and high methylation CpG transitions. All SNVs were included in the analysis and population-specific EM estimates were used.

### Calculating accuracy as a function of genetic distance

To estimate the genetic distance between pairs of genetic variants, we interpolated genetic distances between variant pairs using a genetic map from HapMap2^31^ (https://github.com/joepickrell/1000-genomes-genetic-maps). We downloaded a pre-generated HapMap2 genetic map representing average over recombination rates in the CEU, YRI, and ASN populations. We then ran interpolate_maps.py (downloaded from https://github.com/joepickrell/1000-genomes-genetic-maps/blob/master/scripts/interpolate_maps.py) for all variant pairs in the phasing trio data. As above, accuracy is the proportion of correct classifications.

### MNV analysis

We obtained multi-nucleotide variant pairs for which read-back phasing had previously been calculated^18^. We included only multi-nucleotide variant pairs where each constituent variant was analyzed in our study. Phasing estimates were calculated using cosmopolitan EM estimates.

### Rare disease patient analysis

627 patients from the Broad Institute Center for Mendelian Genetics (CMG)^21^ with a confident or strong candidate genetic diagnosis of a Mendelian condition were selected for analysis. Each patient carried two presumed bi-allelic variants in an autosomal recessive disease gene consistent with the patient’s phenotype. For 293 of the patients, both variants were present in gnomAD and phase was predicted. Trio-sequencing (i.e., sequencing of the proband and the two unaffected biological parents) had been performed for 168 of the 293 patients. For fully sequenced trios, we were able to confirm phasing of the two variants via phase-by-transmission.

### Determining counts of individuals with two rare, damaging variants

Variants were annotated with the worst consequence on the canonical transcript by the Ensembl Variant Effect Predictor (VEP)^32^. Putative LoF variants were annotated with LOFTEE^16^, and only high confidence LoF variants were counted as “pLoF”. Missense variants were annotated with REVEL^22^. REVEL scores ≥0.932 were counted as “strong_revel_missense”, ≥0.773 as “moderate_to_strong_revel_missense”, ≥0.644 as “supporting_to_strong_revel_missense” in line with recent ClinGen recommendations^23^.

Variant pairs were annotated with predicted phase based on the $P_{\text{trans}}$ thresholds. All singleton-singleton variant pairs (AC = 1) and variant pairs with an indeterminate $P_{\text{trans}}$ values (>0.1 and <0.55) were annotated as unphased.

Five allele frequency thresholds were selected for analysis and variant pairs were filtered based on the highest global allele frequency and, where available, the “popmax” allele frequency of each variant in gnomAD (i.e., the highest allele frequency information for the non-bottlenecked population - excluding Ashkenazi Jewish, European Finnish, and “Other” populations): 0.5%, 1%, 1.5%, 2%, and 5%. Further, all variant pairs containing a variant with an allele frequency >5% in a bottlenecked population were filtered out.

The number of individuals carrying a variant pair (irrespective of phase) and the number indicated to be compound heterozygous (in *trans*), unphased (indeterminate), and on the same haplogroup (in *cis*) were counted gene-wise by allele frequency threshold and combined functional consequences (26 consequences). This counting was repeated twice, once restricting individuals to be counted in only one phase group, prioritizing in *trans* over unphased and unphased over in *cis* (displayed in the “variant co-occurrence” gnomAD browser feature), and once allowing individuals to be counted in multiple phase groups, if carrying multiple variant pairs in the same gene with different phase predictions.

### Essential gene lists

The following essential gene lists were queried for the presence of the true “human knock-out” genes identified in this study:

- 2,454 genes essential in mice from Georgi et al. 2013^33^
- 553 pan-cancer core fitness genes from Behan et al., 2019^34^
- 360 core essential genes from genomic perturbation screens from Hart et al. 2014^35^
- 684 genes essential in culture by CRISPR screening from Hart et al. 2017^36^
- 1,075 genes annotated by the ADaM analysis of a large collection of gene dependency profiles (CRISPR-Cas9 screens) across human cancer cell lines from Vinceti et al. 2021^37^

## Supplementary Material

Supplement 1

Supplement 2

Supplement 3

## Data Availability

We provide both web-based look up tools and downloads for the data generated here. A look-up tool to find the likely co-occurrence pattern between two rare (global allele frequency in gnomAD exomes <5%) coding, flanking intronic (from position −1 to −3 in acceptor sites, and +1 to +8 in donor sites) or 5’/3’ UTR variants can be found at: https://gnomad.broadinstitute.org/variant-cooccurrence Additionally, we display the per-gene counts tables that detail the number of individuals with two rare variants, stratified by allele frequency and functional consequence, on each gene’s main page. One table details counts of individuals with two heterozygous variants and includes predicted phase, while the second details individuals with homozygous variants. Both can be found by clicking on the “Variant Co-occurrence” tab on each gene’s main page. All variant co-occurrence tables can be downloaded from: https://gnomad.broadinstitute.org/downloads#v2-variant-cooccurrence

## Genome Aggregation Database Consortium

Maria Abreu^1^, Carlos A. Aguilar Salinas^2^, Tariq Ahmad^3^, Christine M. Albert^4,5^, Jessica Alföldi^6,7^, Diego Ardissino^8^, Irina M. Armean^6,7,9^, Gil Atzmon^10,11^, Eric Banks^12^, John Barnard^13^, Samantha M. Baxter^6^, Laurent Beaugerie^14^, Emelia J. Benjamin^15,16,17^, David Benjamin^12^, Louis Bergelson^12^, Michael Boehnke^18^, Lori L. Bonnycastle^19^, Erwin P. Bottinger^20^, Donald W. Bowden^21,22,23^, Matthew J. Bown^24,25^, Steven Brant^26^, Sarah E. Calvo^6,27^, Hannia Campos^28,29^, John C. Chambers^30,31,32^, Juliana C. Chan^33^, Katherine R. Chao^6^, Sinéad Chapman^6,7,34^, Daniel Chasman^4,35^, Siwei Chen^6,7^, Rex L. Chisholm^36^, Judy Cho^20^, Rajiv Chowdhury^37^, Mina K. Chung^38^, Wendy K. Chung^39,40,41^, Kristian Cibulskis^12^, Bruce Cohen^35,42^, Ryan L. Collins^6,27,43^, Kristen M. Connolly^44^, Adolfo Correa^45^, Miguel Covarrubias^12^, Beryl Cummings^6,43^, Dana Dabelea^46^, Mark J. Daly^6,7,47^, John Danesh^37^, Dawood Darbar^48^, Joshua Denny^49^, Stacey Donnelly^6^, Ravindranath Duggirala^50^, Josée Dupuis^51,52^, Patrick T. Ellinor^6,53^, Roberto Elosua^54,55,56^, James Emery^12^, Eleina England^6^, Jeanette Erdmann^57,58,59^, Tõnu Esko^6,60^, Emily Evangelista^6^, Yossi Farjoun^12^, Diane Fatkin^61,62,63^, Steven Ferriera^64^, Jose Florez^35,65,66^, Laurent C. Francioli^6,7^, Andre Franke^67^, Martti Färkkilä^68^, Stacey Gabriel^64^, Kiran Garimella^12^, Laura D. Gauthier^12^, Jeff Gentry^12^, Gad Getz^35,69,70^, David C. Glahn^71,72^, Benjamin Glaser^73^, Stephen J. Glatt^74^, David Goldstein^75,76^, Clicerio Gonzalez^77^, Julia K. Goodrich^6^, Leif Groop^78,79^, Sanna Gudmundsson^6,7,80^, Namrata Gupta^6,64^, Andrea Haessly^12^, Christopher Haiman^81^, Ira Hall^82^, Craig Hanis^83^, Matthew Harms^84,85^, Mikko Hiltunen^86^, Matti M. Holi^87^, Christina M. Hultman^88,89^, Chaim Jalas^90^, Thibault Jeandet^12^, Mikko Kallela^91^, Diane Kaplan^12^, Jaakko Kaprio^79^, Konrad J. Karczewski^6,7,34^, Sekar Kathiresan^27,35,92^, Eimear Kenny^89,93^, Bong-Jo Kim^94^, Young Jin Kim^94^, George Kirov^95^, Zan Koenig^6^, Jaspal Kooner^31,96,97^, Seppo Koskinen^98^, Harlan M. Krumholz^99^, Subra Kugathasan^100^, Soo Heon Kwak^101^, Markku Laakso^102,103^, Nicole Lake^104^, Trevyn Langsford^12^, Kristen M. Laricchia^6,7^, Terho Lehtimäki^105^, Monkol Lek^104^, Emily Lipscomb^6^, Christopher Llanwarne^12^, Ruth J.F. Loos^20,106^, Steven A. Lubitz^6,53^, Teresa Tusie Luna^107,108^, Ronald C.W. Ma^33,109,110^, Daniel G. MacArthur^6,111,112^, Gregory M. Marcus^113^, Jaume Marrugat^55,114^, Alicia R. Martin^6^, Kari M. Mattila^105^, Steven McCarroll^34,115^, Mark I. McCarthy^116,117,118^, Jacob McCauley^119,120^, Dermot McGovern^121^, Ruth McPherson^122^, James B. Meigs^6,35,123^, Olle Melander^124^, Andres Metspalu^125^, Deborah Meyers^126^, Eric V. Minikel^6^, Braxton D. Mitchell^127^, Vamsi K. Mootha^6,128^, Ruchi Munshi^12^, Aliya Naheed^129^, Saman Nazarian^130,131^, Benjamin M. Neale^6,7^, Peter M. Nilsson^132^, Sam Novod^12^, Anne H. O’Donnell-Luria^6,7,80^, Michael C. O’Donovan^95^, Yukinori Okada^133,134,135^, Dost Ongur^35,42^, Lorena Orozco^136^, Michael J. Owen^95^, Colin Palmer^137^, Nicholette D. Palmer^138^, Aarno Palotie^7,34,79^, Kyong Soo Park^101,139^, Carlos Pato^140^, Nikelle Petrillo^12^, William Phu^6,80^, Timothy Poterba^6,7,34^, Ann E. Pulver^141^, Dan Rader^130,142^, Nazneen Rahman^143^, Heidi L. Rehm^6,27^, Alex Reiner^144,145^, Anne M. Remes^146^, Dan Rhodes^6^, Stephen Rich^147,148^, John D. Rioux^149,150^, Samuli Ripatti^79,151,152^, David Roazen^12^, Dan M. Roden^153,154^, Jerome I. Rotter^155^, Valentin Ruano-Rubio^12^, Nareh Sahakian^12^, Danish Saleheen^156,157,158^, Veikko Salomaa^159^, Andrea Saltzman^6^, Nilesh J. Samani^24,25^, Kaitlin E. Samocha^6,7,27^, Jeremiah Scharf^6,27,34^, Molly Schleicher^6^, Heribert Schunkert^160,161^, Sebastian Schönherr^162^, Eleanor Seaby^6^, Cotton Seed^7,34^, Svati H. Shah^163^, Megan Shand^12^, Moore B. Shoemaker^164^, Tai Shyong^165,166^, Edwin K. Silverman^167,168^, Moriel Singer-Berk^6^, Pamela Sklar^169,170,171^, J. Gustav Smith^152,172,173^, Jonathan T. Smith^12^, Hilkka Soininen^174^, Harry Sokol^175,176,177^, Matthew Solomonson^6,7^, Rachel G. Son^6^, Jose Soto^12^, Tim Spector^178^, Christine Stevens^6,7,34^, Nathan Stitziel^82,179^, Patrick F. Sullivan^88,180^, Jaana Suvisaari^159^, E. Shyong Tai^181,182,183^, Michael E. Talkowski^6,27,34^, Yekaterina Tarasova^6^, Kent D. Taylor^155^, Yik Ying Teo^181,184,185^, Grace Tiao^6,7^, Kathleen Tibbetts^12^, Charlotte Tolonen^12^, Ming Tsuang^186,187^, Tiinamaija Tuomi^79,188,189^, Dan Turner^190^, Teresa Tusie-Luna^191,192^, Erkki Vartiainen^193^, Marquis Vawter^194^, Christopher Vittal^6,7^, Gordon Wade^12^, Arcturus Wang^6,7,34^, Qingbo Wang^6,133^, James S. Ware^6,195,196^, Hugh Watkins^197^, Nicholas A. Watts^6,7^, Rinse K. Weersma^198^, Ben Weisburd^12^, Maija Wessman^79,199^, Nicola Whiffin^6,200,201^, Michael W. Wilson^6,7^, James G. Wilson^202^, Ramnik J. Xavier^203,204^, Mary T. Yohannes^6^

^1^University of Miami Miller School of Medicine, Gastroenterology, Miami, USA

^2^Unidad de Investigacion de Enfermedades Metabolicas, Instituto Nacional de Ciencias Medicas y Nutricion, Mexico City, Mexico

^3^Peninsula College of Medicine and Dentistry, Exeter, UK

^4^Division of Preventive Medicine, Brigham and Women’s Hospital, Boston, MA, USA

^5^Division of Cardiovascular Medicine, Brigham and Women’s Hospital and Harvard Medical School, Boston, MA, USA

^6^Program in Medical and Population Genetics, Broad Institute of MIT and Harvard, Cambridge, MA, USA

^7^Analytic and Translational Genetics Unit, Massachusetts General Hospital, Boston, MA, USA

^8^Department of Cardiology University Hospital, Parma, Italy

^9^European Molecular Biology Laboratory, European Bioinformatics Institute, Wellcome Genome Campus, Hinxton, Cambridge, UK

^10^Department of Biology Faculty of Natural Sciences, University of Haifa, Haifa, Israel

^11^Departments of Medicine and Genetics, Albert Einstein College of Medicine, Bronx, NY, USA

^12^Data Science Platform, Broad Institute of MIT and Harvard, Cambridge, MA, USA

^13^Department of Quantitative Health Sciences, Lerner Research Institute Cleveland Clinic, Cleveland, OH, USA

^14^Sorbonne Université, APHP, Gastroenterology Department Saint Antoine Hospital, Paris, France

^15^NHLBI and Boston University’s Framingham Heart Study, Framingham, MA, USA

^16^Department of Medicine, Boston University Chobanian and Avedisian School of Medicine, Boston, MA, USA

^17^Department of Epidemiology, Boston University School of Public Health, Boston, MA, USA

^18^Department of Biostatistics and Center for Statistical Genetics, University of Michigan, Ann Arbor, MI, USA

^19^National Human Genome Research Institute, National Institutes of Health Bethesda, MD, USA

^20^The Charles Bronfman Institute for Personalized Medicine, Icahn School of Medicine at Mount Sinai, New York, NY, USA

^21^Department of Biochemistry, Wake Forest School of Medicine, Winston-Salem, NC, USA

^22^Center for Genomics and Personalized Medicine Research, Wake Forest School of Medicine, Winston-Salem, NC, USA

^23^Center for Diabetes Research, Wake Forest School of Medicine, Winston-Salem, NC, USA

^24^Department of Cardiovascular Sciences, University of Leicester, Leicester, UK

^25^NIHR Leicester Biomedical Research Centre, Glenfield Hospital, Leicester, UK

^26^John Hopkins Bloomberg School of Public Health, Baltimore, MD, USA

^27^Center for Genomic Medicine, Massachusetts General Hospital, Boston, MA, USA

^28^Harvard School of Public Health, Boston, MA, USA

^29^Central American Population Center, San Pedro, Costa Rica

^30^Department of Epidemiology and Biostatistics, Imperial College London, London, UK

^31^Department of Cardiology, Ealing Hospital, NHS Trust, Southall, UK

^32^Imperial College, Healthcare NHS Trust Imperial College London, London, UK

^33^Department of Medicine and Therapeutics, The Chinese University of Hong Kong, Hong Kong, China

^34^Stanley Center for Psychiatric Research, Broad Institute of MIT and Harvard, Cambridge, MA, USA

^35^Department of Medicine, Harvard Medical School, Boston, MA, USA

^36^Northwestern University Feinberg School of Medicine, Chicago, IL, USA

^37^University of Cambridge, Cambridge, England

^38^Departments of Cardiovascular, Medicine Cellular and Molecular Medicine Molecular Cardiology, Quantitative Health Sciences, Cleveland Clinic, Cleveland, OH, USA

^39^Department of Pediatrics, Columbia University Irving Medical Center, New York, NY, USA

^40^Herbert Irving Comprehensive Cancer Center, Columbia University Medical Center, New York, NY, USA

^41^Department of Medicine, Columbia University Medical Center, New York, NY, USA

^42^McLean Hospital, Belmont, MA, USA

^43^Division of Medical Sciences, Harvard Medical School, Boston, MA, USA

^44^Genomics Platform, Broad Institute of MIT and Harvard, Cambridge, MA, USA

^45^Department of Medicine, University of Mississippi Medical Center, Jackson, MI, USA

^46^Department of Epidemiology Colorado School of Public Health Aurora, CO, USA

^47^Institute for Molecular Medicine Finland, (FIMM) Helsinki, Finland

^48^Department of Medicine and Pharmacology, University of Illinois at Chicago, Chicago, IL, USA

^49^Vanderbilt University Medical Center, Nashville, TN, USA

^50^Department of Genetics, Texas Biomedical Research Institute, San Antonio, TX, USA

^51^Department of Biostatistics, Boston University School of Public Health, Boston, MA, USA

^52^National Heart Lung and Blood Institute’s Framingham Heart Study, Framingham, MA, USA

^53^Cardiac Arrhythmia Service and Cardiovascular Research Center, Massachusetts General Hospital, Boston, MA, USA

^54^Cardiovascular Epidemiology and Genetics Hospital del Mar Medical Research Institute, (IMIM) Barcelona Catalonia, Spain

^55^CIBER CV Barcelona, Catalonia, Spain

^56^Departament of Medicine, Medical School University of Vic-Central, University of Catalonia, Vic Catalonia, Spain

^57^Institute for Cardiogenetics, University of Lübeck, Lübeck, Germany

^58^German Research Centre for Cardiovascular Research, Hamburg/Lübeck/Kiel, Lübeck, Germany

^59^University Heart Center Lübeck, Lübeck, Germany

^60^Estonian Genome Center, Institute of Genomics University of Tartu, Tartu, Estonia

^61^Victor Chang Cardiac Research Institute, Darlinghurst, NSW, Australia

^62^Faculty of Medicine, UNSW Sydney, Kensington, NSW, Australia

^63^Cardiology Department, St Vincent’s Hospital, Darlinghurst, NSW, Australia

^64^Broad Genomics, Broad Institute of MIT and Harvard, Cambridge, MA, USA

^65^Diabetes Unit and Center for Genomic Medicine, Massachusetts General Hospital, Boston, MA, USA

^66^Programs in Metabolism and Medical & Population Genetics, Broad Institute of MIT and Harvard, Cambridge, MA, USA

^67^Institute of Clinical Molecular Biology, (IKMB) Christian-Albrechts-University of Kiel, Kiel, Germany

^68^Helsinki University and Helsinki University Hospital Clinic of Gastroenterology, Helsinki, Finland

^69^Bioinformatics Program MGH Cancer Center and Department of Pathology, Boston, MA, USA

^70^Cancer Genome Computational Analysis, Broad Institute of MIT and Harvard, Cambridge, MA, USA

^71^Department of Psychiatry and Behavioral Sciences, Boston Children’s Hospitaland Harvard Medical School, Boston, MA, USA

^72^Harvard Medical School Teaching Hospital, Boston, MA, USA

^73^Department of Endocrinology and Metabolism, Hadassah Medical Center and Faculty of Medicine, Hebrew University of Jerusalem, Israel

^74^Department of Psychiatry and Behavioral Sciences, SUNY Upstate Medical University, Syracuse, NY, USA

^75^Institute for Genomic Medicine, Columbia University Medical Center Hammer Health Sciences, New York, NY, USA

^76^Department of Genetics & Development Columbia University Medical Center, Hammer Health Sciences, New York, NY, USA

^77^Centro de Investigacion en Salud Poblacional, Instituto Nacional de Salud Publica, Mexico

^78^Lund University Sweden, Sweden

^79^Institute for Molecular Medicine Finland, (FIMM) HiLIFE University of Helsinki, Helsinki, Finland

^80^Division of Genetics and Genomics, Boston Children’s Hospital, Boston, MA, USA

^81^Lund University Diabetes Centre, Malmö, Skåne County, Sweden

^82^Washington School of Medicine, St Louis, MI, USA

^83^Human Genetics Center University of Texas Health Science Center at Houston, Houston, TX, USA

^84^Department of Neurology Columbia University, New York City, NY, USA

^85^Institute of Genomic Medicine, Columbia University, New York City, NY, USA

^86^Institute of Biomedicine, University of Eastern Finland, Kuopio, Finland

^87^Department of Psychiatry, Helsinki University Central Hospital Lapinlahdentie, Helsinki, Finland

^88^Department of Medical Epidemiology and Biostatistics, Karolinska Institutet, Stockholm, Sweden

^89^Icahn School of Medicine at Mount Sinai, New York, NY, USA

^90^Bonei Olam, Center for Rare Jewish Genetic Diseases, Brooklyn, NY, USA

^91^Department of Neurology, Helsinki University, Central Hospital, Helsinki, Finland

^92^Cardiovascular Disease Initiative and Program in Medical and Population Genetics, Broad Institute of MIT and Harvard, Cambridge, MA, USA

^93^Charles Bronfman Institute for Personalized Medicine, New York, NY, USA

^94^Division of Genome Science, Department of Precision Medicine, National Institute of Health, Republic of Korea

^95^MRC Centre for Neuropsychiatric Genetics & Genomics, Cardiff University School of Medicine, Cardiff, Wales

^96^Imperial College, Healthcare NHS Trust, London, UK

^97^National Heart and Lung Institute Cardiovascular Sciences, Hammersmith Campus, Imperial College London, London, UK

^98^Department of Health THL-National Institute for Health and Welfare, Helsinki, Finland

^99^Section of Cardiovascular Medicine, Department of Internal Medicine, Yale School of Medicine, Center for Outcomes Research and Evaluation Yale-New Haven Hospital, New Haven, CT, USA

^100^Division of Pediatric Gastroenterology, Emory University School of Medicine, Atlanta, GA, USA

^101^Department of Internal Medicine, Seoul National University Hospital, Seoul, Republic of Korea

^102^The University of Eastern Finland, Institute of Clinical Medicine, Kuopio, Finland

^103^Kuopio University Hospital, Kuopio, Finland

^104^Department of Genetics, Yale School of Medicine, New Haven, CT, USA

^105^Department of Clinical Chemistry Fimlab Laboratories and Finnish Cardiovascular Research Center-Tampere Faculty of Medicine and Health Technology, Tampere University, Finland

^106^The Mindich Child Health and Development, Institute Icahn School of Medicine at Mount Sinai, New York, NY, USA

^107^National Autonomous University of Mexico, Mexico City, Mexico

^108^Salvador Zubirán National Institute of Health Sciences and Nutrition, Mexico City, Mexico

^109^Li Ka Shing Institute of Health Sciences, The Chinese University of Hong Kong, Hong Kong, China

^110^Hong Kong Institute of Diabetes and Obesity, The Chinese University of Hong Kong, Hong Kong, China

^111^Centre for Population Genomics, Garvan Institute of Medical Research and UNSW Sydney, Sydney, Australia

^112^Centre for Population Genomics, Murdoch Children’s Research Institute, Melbourne, Australia

^113^University of California San Francisco Parnassus Campus, San Francisco, CA, USA

^114^Cardiovascular Research REGICOR Group, Hospital del Mar Medical Research Institute, (IMIM) Barcelona, Catalonia, Spain

^115^Department of Genetics, Harvard Medical School, Boston, MA, USA

^116^Oxford Centre for Diabetes, Endocrinology and Metabolism, University of Oxford, Churchill Hospital Old Road Headington, Oxford, OX, LJ, UK

^117^Welcome Centre for Human Genetics, University of Oxford, Oxford, OX, BN, UK

^118^Oxford NIHR Biomedical Research Centre, Oxford University Hospitals, NHS Foundation Trust, John Radcliffe Hospital, Oxford, OX, DU, UK

^119^John P. Hussman Institute for Human Genomics, Leonard M. Miller School of Medicine, University of Miami, Miami, FL, USA

^120^The Dr. John T. Macdonald Foundation Department of Human Genetics, Leonard M. Miller School of Medicine, University of Miami, Miami, FL, USA

^121^F. Widjaja Foundation Inflammatory Bowel and Immunobiology Research Institute Cedars-Sinai Medical Center, Los Angeles, CA, USA

^122^Atherogenomics Laboratory University of Ottawa, Heart Institute, Ottawa, Canada

^123^Division of General Internal Medicine, Massachusetts General Hospital, Boston, MA, USA

^124^Department of Clinical Sciences University, Hospital Malmo Clinical Research Center, Lund University, Malmö, Sweden

^125^Estonian Genome Center, Institute of Genomics, University of Tartu, Tartu, Estonia

^126^University of Arizona Health Science, Tuscon, AZ, USA

^127^Department of Medicine, University of Maryland School of Medicine, Baltimore, MD, USA

^128^Howard Hughes Medical Institute and Department of Molecular Biology, Massachusetts General Hospital, Boston, MA, USA

^129^International Centre for Diarrhoeal Disease Research, Bangladesh

^130^Perelman School of Medicine, University of Pennsylvania, Philadelphia, PA, USA

^131^Johns Hopkins Bloomberg School of Public Health, Baltimore, MD, USA

^132^Lund University, Dept. Clinical Sciences, Skåne University Hospital, Malmö, Sweden

^133^Department of Statistical Genetics, Osaka University Graduate School of Medicine, Suita, Japan

^134^Laboratory of Statistical Immunology, Immunology Frontier Research Center (WPI-IFReC), Osaka University, Suita, Japan

^135^Integrated Frontier Research for Medical Science Division, Institute for Open and Transdisciplinary Research Initiatives, Osaka University, Suita, Japan

^136^Instituto Nacional de Medicina Genómica, (INMEGEN) Mexico City, Mexico

^137^Medical Research Institute, Ninewells Hospital and Medical School University of Dundee, Dundee, UK

^138^Wake Forest School of Medicine, Winston-Salem, NC, USA

^139^Department of Molecular Medicine and Biopharmaceutical Sciences, Graduate School of Convergence Science and Technology, Seoul National University, Seoul, Republic of Korea

^140^Department of Psychiatry Keck School of Medicine at the University of Southern California, Los Angeles, CA, USA

^141^Department of Psychiatry and Behavioral Sciences, Johns Hopkins University School of Medicine, Baltimore, MD, USA

^142^Children’s Hospital of Philadelphia, Philadelphia, PA, USA

^143^Division of Genetics and Epidemiology, Institute of Cancer Research, London, SM, NG

^144^University of Washington, Seattle, WA, USA

^145^Fred Hutchinson Cancer Research Center, Seattle, WA, USA

^146^Medical Research Center, Oulu University Hospital, Oulu Finland and Research Unit of Clinical Neuroscience Neurology University of Oulu, Oulu, Finland

^147^Center for Public Health Genomics, University of Virginia, Charlottesville, VA, USA

^148^Department of Public Health Sciences, University of Virginia, Charlottesville, VA, USA

^149^Research Center Montreal Heart Institute, Montreal, Quebec, Canada

^150^Department of Medicine, Faculty of Medicine Université de Montréal, Québec, Canada

^151^Department of Public Health Faculty of Medicine, University of Helsinki, Helsinki, Finland

^152^Broad Institute of MIT and Harvard, Cambridge, MA, USA

^153^Department of Biomedical Informatics Vanderbilt, University Medical Center, Nashville, TN, USA

^154^Department of Medicine, Vanderbilt University Medical Center, Nashville, TN, USA

^155^The Institute for Translational Genomics and Population Sciences, Department of Pediatrics, The Lundquist Institute for Biomedical Innovation at Harbor-UCLA Medical Center, Torrance, CA, USA

^156^Department of Biostatistics and Epidemiology, Perelman School of Medicine, University of Pennsylvania, Philadelphia, PA, USA

^157^Department of Medicine, Perelman School of Medicine at the University of Pennsylvania, Philadelphia, PA, USA

^158^Center for Non-Communicable Diseases, Karachi, Pakistan

^159^National Institute for Health and Welfare, Helsinki, Finland

^160^Deutsches Herzzentrum, München, Germany

^161^Technische Universität München, Germany

^162^Institute of Genetic Epidemiology, Department of Genetics and Pharmacology, Medical University of Innsbruck, 6020 Innsbruck, Austria

^163^Duke Molecular Physiology Institute, Durham, NC

^164^Division of Cardiovascular Medicine, Nashville VA Medical Center, Vanderbilt University School of Medicine, Nashville, TN, USA

^165^Division of Endocrinology, National University Hospital, Singapore

^166^NUS Saw Swee Hock School of Public Health, Singapore

^167^Channing Division of Network Medicine, Brigham and Women’s Hospital, Boston, MA, USA

^168^Harvard Medical School, Boston, MA, USA

^169^Department of Psychiatry, Icahn School of Medicine at Mount Sinai, New York, NY, USA

^170^Department of Genetics and Genomic Sciences, Icahn School of Medicine at Mount Sinai, New York, NY, USA

^171^Institute for Genomics and Multiscale Biology, Icahn School of Medicine at Mount Sinai, New York, NY, USA

^172^The Wallenberg Laboratory/Department of Molecular and Clinical Medicine, Institute of Medicine, Gothenburg University and the Department of Cardiology, Sahlgrenska University Hospital, Gothenburg, Sweden

^173^Department of Cardiology, Wallenberg Center for Molecular Medicine and Lund University Diabetes Center, Clinical Sciences, Lund University and Skåne University Hospital, Lund, Sweden

^174^Institute of Clinical Medicine Neurology, University of Eastern Finad, Kuopio, Finland

^175^Sorbonne Université, INSERM, Centre de Recherche Saint-Antoine, CRSA, AP-HP, Saint Antoine Hospital, Gastroenterology department, F-75012 Paris, France

^176^INRA, UMR1319 Micalis & AgroParisTech, Jouy en Josas, France

^177^Paris Center for Microbiome Medicine, (PaCeMM) FHU, Paris, France

^178^Department of Twin Research and Genetic Epidemiology King’s College London, London, UK

^179^The McDonnell Genome Institute at Washington University, Seattle, WA, USA

^180^Departments of Genetics and Psychiatry, University of North Carolina, Chapel Hill, NC, USA

^181^Saw Swee Hock School of Public Health National University of Singapore, National University Health System, Singapore

^182^Department of Medicine, Yong Loo Lin School of Medicine National University of Singapore, Singapore

^183^Duke-NUS Graduate Medical School, Singapore

^184^Life Sciences Institute, National University of Singapore, Singapore

^185^Department of Statistics and Applied Probability, National University of Singapore, Singapore

^186^Center for Behavioral Genomics, Department of Psychiatry, University of California, San Diego, CA, USA

^187^Institute of Genomic Medicine, University of California San Diego, San Diego, CA, USA

^188^Endocrinology, Abdominal Center, Helsinki University Hospital, Helsinki, Finland

^189^Institute of Genetics, Folkhalsan Research Center, Helsinki, Finland

^190^Juliet Keidan Institute of Pediatric Gastroenterology Shaare Zedek Medical Center, The Hebrew University of Jerusalem, Jerusalem, Israel

^191^Instituto de Investigaciones Biomédicas, UNAM, Mexico City, Mexico

^192^Instituto Nacional de Ciencias Médicas y Nutrición Salvador Zubirán, Mexico City, Mexico

^193^Department of Public Health Faculty of Medicine University of Helsinki, Helsinki, Finland

^194^Department of Psychiatry and Human Behavior, University of California Irvine, Irvine, CA, USA

^195^National Heart & Lung Institute & MRC London Institute of Medical Sciences, Imperial College, London, UK

^196^Royal Brompton & Harefield Hospitals, Guy’s and St. Thomas’ NHS Foundation Trust, London, UK

^197^Radcliffe Department of Medicine, University of Oxford, Oxford, UK

^198^Department of Gastroenterology and Hepatology, University of Groningen and University Medical Center Groningen, Groningen, Netherlands

^199^Folkhälsan Institute of Genetics, Folkhälsan Research Center, Helsinki, Finland

^200^National Heart & Lung Institute and MRC London Institute of Medical Sciences, Imperial College London, London, UK

^201^Cardiovascular Research Centre, Royal Brompton & Harefield Hospitals NHS Trust, London, UK

^202^Department of Physiology and Biophysics, University of Mississippi Medical Center, Jackson, MS, USA

^203^Program in Infectious Disease and Microbiome, Broad Institute of MIT and Harvard, Cambridge, MA, USA

^204^Center for Computational and Integrative Biology, Massachusetts General Hospital, Boston, MA, USA

Authors received funding as follows:

Emelia J Benjamin: Framingham Heart Study 75N92019D00031; and R01HL092577

Matthew J. Bown: British Heart Foundation awards CS/14/2/30841 and RG/18/10/33842

Josée Dupuis: National Heart Lung and Blood Institute’s Framingham Heart Study Contract (HHSNI); National Institute for Diabetes and Digestive and Kidney Diseases (NIDDK) R DK

Martti Färkkilä: State funding for university level health research

Laura D. Gauthier: Intel, Illumina

Stephen J. Glatt: U.S. NIMH Grant R MH

Leif Groop: The Academy of Finland and University of Helsinki: Center of Excellence for Complex Disease Genetics (grant number 312063 and 336822), Sigrid Jusélius Foundation; IMI 2 (grant No 115974 and 15881)

Mikko Hiltunen: Academy of Finland (grant 338182) Sigrid Jusélius Foundation the Strategic Neuroscience Funding of the University of Eastern Finland

Chaim Jalas: Bonei Olam

Jaakko Kaprio: Academy of Finland (grants 312073 and 336823)

Jacob McCauley: National Institute of Diabetes and Digestive and Kidney Disease Grant R01DK104844

Yukinori Okada: JSPS KAKENHI (19H01021, 20K21834), AMED (JP21km0405211, JP21ek0109413, JP21gm4010006, JP21km0405217, JP21ek0410075), JST Moonshot R&D (JPMJMS2021)

Michael J. Owen: Medical Research Council UK: Centre Grant No. MR/L010305/1, Program Grant No. G0800509

Aarno Palotie: the Academy of Finland Center of Excellence for Complex Disease Genetics (grant numbers 312074 and 336824) and Sigrid Jusélius Foundation

John D. Rioux: National Institute of Diabetes and Digestive and Kidney Diseases (NIDDK; DK062432), from the Canadian Institutes of Health (CIHR GPG 102170), from Genome Canada/Génome Québec (GPH-129341), and a Canada Research Chair (#230625)

Samuli Ripatti: the Academy of Finland Center of Excellence for Complex Disease Genetics (grant number ) Sigrid Jusélius Foundation

Jerome I. Rotter: Trans-Omics in Precision Medicine (TOPMed) program was supported by the National Heart, Lung and Blood Institute (NHLBI). WGS for “NHLBI TOPMed: Multi-Ethnic Study of Atherosclerosis (MESA)” (phs001416.v1.p1) was performed at the Broad Institute of MIT and Harvard (3U54HG003067-13S1). Core support including centralized genomic read mapping and genotype calling, along with variant quality metrics and filtering were provided by the TOPMed Informatics Research Center (3R01HL-117626-02S1; contract HHSN268201800002I). Core support including phenotype harmonization, data management, sample-identity QC, and general program coordination were provided by the TOPMed Data Coordinating Center (R01HL-120393; U01HL-120393; contract HHSN268201800001I). We gratefully acknowledge the studies and participants who provided biological samples and data for MESA and TOPMed. JSK was supported by the Pulmonary Fibrosis Foundation Scholars Award and grant K23-HL-150301 from the NHLBI. MRA was supported by grant K23-HL-150280, AJP was supported by grant K23-HL-140199, and AM was supported by R01-HL131565 from the NHLBI. EJB was supported by grant K23-AR-075112 from the National Institute of Arthritis and Musculoskeletal and Skin Diseases. The MESA project is conducted and supported by the National Heart, Lung, and Blood Institute (NHLBI) in collaboration with MESA investigators. Support for MESA is provided by contracts 75N92020D00001, HHSN268201500003I, N01-HC-95159, 75N92020D00005, N01-HC-95160, 75N92020D00002, N01-HC-95161, 75N92020D00003, N01-HC-95162, 75N92020D00006, N01-HC-95163, 75N92020D00004, N01-HC-95164, 75N92020D00007, N01-HC-95165, N01-HC-95166, N01-HC-95167, N01-HC-95168, N01-HC-95169, UL1-TR-000040, UL1-TR-001079, and UL1-TR-001420. Also supported in part by the National Center for Advancing Translational Sciences, CTSI grant UL1TR001881, and the National Institute of Diabetes and Digestive and Kidney Disease Diabetes Research Center (DRC) grant DK063491 to the Southern California Diabetes Endocrinology Research Center

Edwin K. Silverman: NIH Grants U01 HL089856 and U01 HL089897

J. Gustav Smith: The Swedish Heart-Lung Foundation (2019–0526), the Swedish Research Council (2017-02554), the European Research Council (ERC-STG-2015-679242), Skåne University Hospital, governmental funding of clinical research within the Swedish National Health Service, a generous donation from the Knut and Alice Wallenberg foundation to the Wallenberg Center for Molecular Medicine in Lund, and funding from the Swedish Research Council (Linnaeus grant Dnr 349-2006-237, Strategic Research Area Exodiab Dnr 2009–1039) and Swedish Foundation for Strategic Research (Dnr IRC15-0067) to the Lund University Diabetes Center

Kent D. Taylor: Trans-Omics in Precision Medicine (TOPMed) program was supported by the National Heart, Lung and Blood Institute (NHLBI). WGS for “NHLBI TOPMed: Multi-Ethnic Study of Atherosclerosis (MESA)” (phs001416.v1.p1) was performed at the Broad Institute of MIT and Harvard (3U54HG003067-13S1). Core support including centralized genomic read mapping and genotype calling, along with variant quality metrics and filtering were provided by the TOPMed Informatics Research Center (3R01HL-117626-02S1; contract HHSN268201800002I). Core support including phenotype harmonization, data management, sample-identity QC, and general program coordination were provided by the TOPMed Data Coordinating Center (R01HL-120393; U01HL-120393; contract HHSN268201800001I). We gratefully acknowledge the studies and participants who provided biological samples and data for MESA and TOPMed. JSK was supported by the Pulmonary Fibrosis Foundation Scholars Award and grant K23-HL-150301 from the NHLBI. MRA was supported by grant K23-HL-150280, AJP was supported by grant K23-HL-140199, and AM was supported by R01-HL131565 from the NHLBI. EJB was supported by grant K23-AR-075112 from the National Institute of Arthritis and Musculoskeletal and Skin Diseases. The MESA project is conducted and supported by the National Heart, Lung, and Blood Institute (NHLBI) in collaboration with MESA investigators. Support for MESA is provided by contracts 75N92020D00001, HHSN268201500003I, N01-HC-95159, 75N92020D00005, N01-HC-95160, 75N92020D00002, N01-HC-95161, 75N92020D00003, N01-HC-95162, 75N92020D00006, N01-HC-95163, 75N92020D00004, N01-HC-95164, 75N92020D00007, N01-HC-95165, N01-HC-95166, N01-HC-95167, N01-HC-95168, N01-HC-95169, UL1-TR-000040, UL1-TR-001079, and UL1-TR-001420. Also supported in part by the National Center for Advancing Translational Sciences, CTSI grant UL1TR001881, and the National Institute of Diabetes and Digestive and Kidney Disease Diabetes Research Center (DRC) grant DK063491 to the Southern California Diabetes Endocrinology Research Center

Tiinamaija Tuomi: The Academy of Finland and University of Helsinki: Center of Excellence for Complex Disease Genetics (grant number 312072 and 336826 ), Folkhalsan Research Foundation, Helsinki University Hospital, Ollqvist Foundation, Liv och Halsa foundation; NovoNordisk Foundation

Teresa Tusie-Luna: CONACyT Project 312688

James S. Ware: Sir Jules Thorn Charitable Trust [21JTA], Wellcome Trust [107469/Z/15/Z], Medical Research Council (UK), NIHR Imperial College Biomedical Research Centre

Rinse K. Weersma: The Lifelines Biobank initiative has been made possible by subsidy from the Dutch Ministry of Health Welfare and Sport the Dutch Ministry of Economic Affairs the University Medical Centre Groningen (UMCG the Netherlands ) the University of Groningen and the Northern Provinces of the Netherlands

No conflicts of interest to declare
