# Supplementary material for: Inferring compound heterozygosity from large-scale exome sequencing data
Source: bioRxiv. 2023 Aug 21:2023.03.19.533370. Originally published 2023 Mar 23. Preprint. [Version 2] doi: 10.1101/2023.03.19.533370 (PMC10055215; doi:10.1101/2023.03.19.533370)
Supplement: Supplement 3 [file NIHPP2023.03.19.533370v2-supplement-3.pdf]

bioRxiv preprint doi: https://doi.org/10.1101/2023.03.19.533370; this version posted August 21, 2023. The copyright holder for this preprint (which was not certified by peer review) is the author/funder, who has granted bioRxiv a license to display the preprint in perpetuity. It is made available under aCC-BY 4.0 International license.

Supplementary Figures

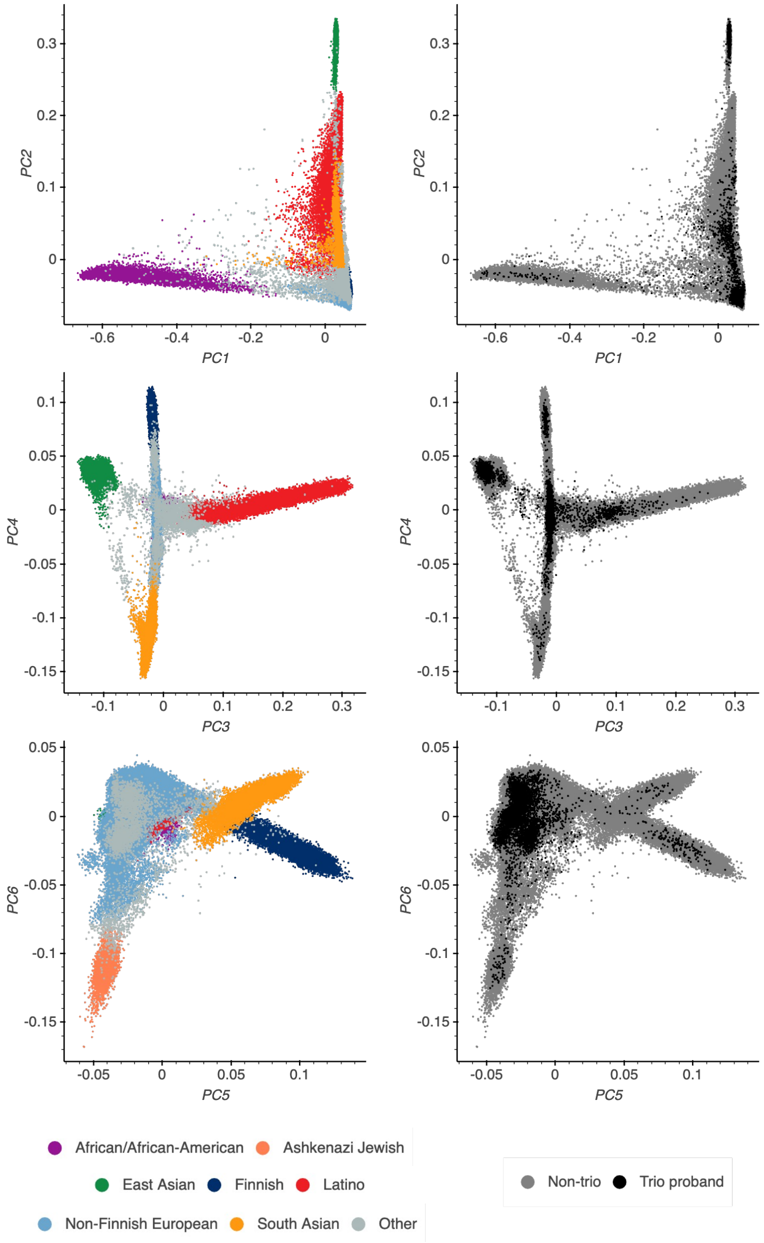

Supplementary Figure 1: Principal component analysis (PCA) plot for the full gnomAD v2 cohort (left) and specifically for the trios (right, trios in black) included in this paper. The top row shows PC1 vs PC2, the middle row shows PC3 vs PC4, and the bottom row shows PC5 vs PC6. Genetic ancestry group labels for the global gnomAD populations were done as described in Karczewski et al. 2020[20].

bioRxiv preprint doi: https://doi.org/10.1101/2023.03.19.533370; this version posted August 21, 2023. The copyright holder for this preprint (which was not certified by peer review) is the author/funder, who has granted bioRxiv a license to display the preprint in perpetuity. It is made available under aCC-BY 4.0 International license.

Figure S2

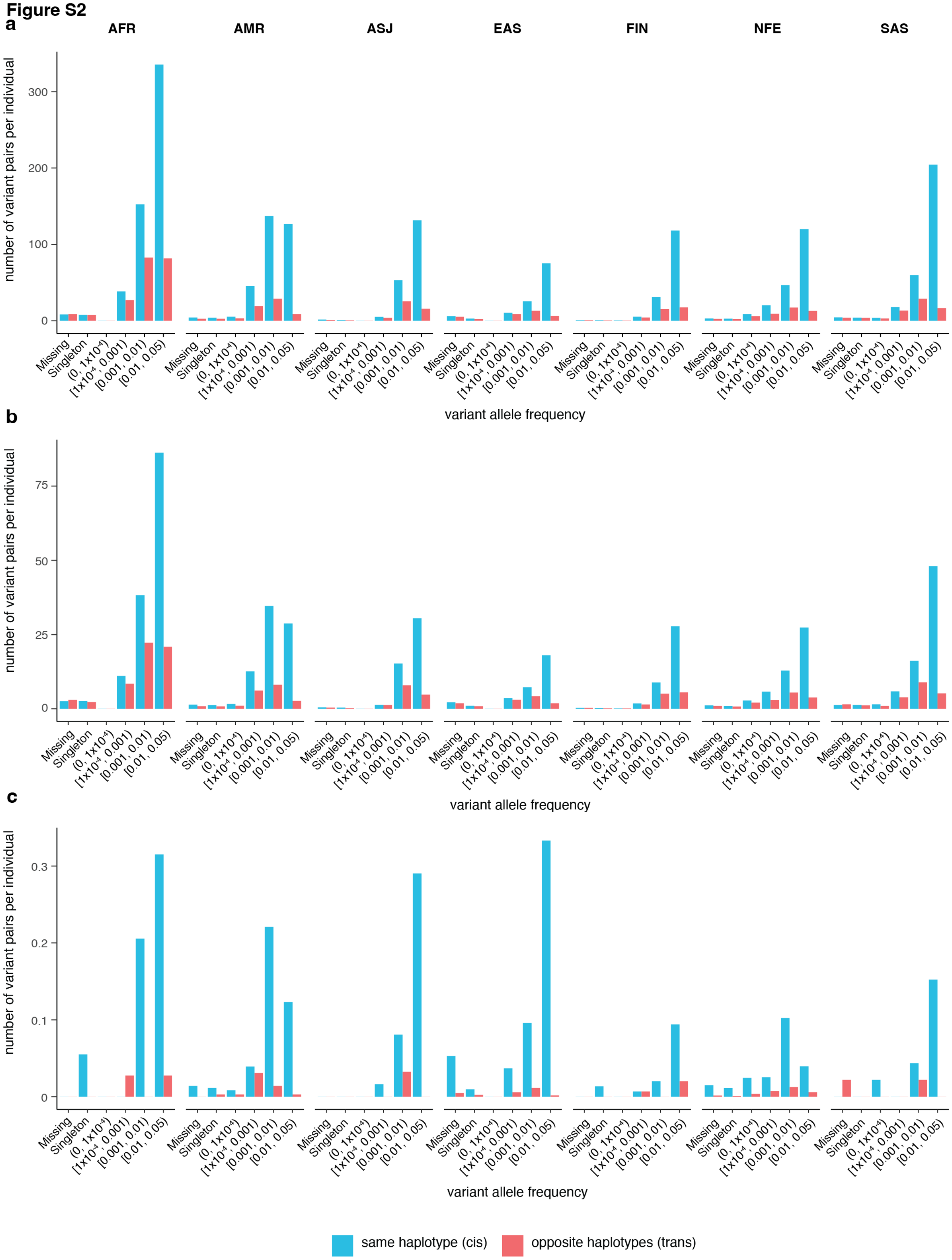

bioRxiv preprint doi: https://doi.org/10.1101/2023.03.19.533370; this version posted August 21, 2023. The copyright holder for this preprint (which was not certified by peer review) is the author/funder, who has granted bioRxiv a license to display the preprint in perpetuity. It is made available under aCC-BY 4.0 International license.

Supplementary Figure 2: Number of variant pairs observed per trio sample as a function of ancestry and AF. All variant pairs are shown in **a.** Variant pairs in which both variants are moderate effect or predicted loss-of-function (pLoF) are shown in **b.** Variant pairs in which both variants are pLoF are shown in **c.** Variant AF is the AF of the less common variant in a given variant pair and is population-specific frequency. AFR = African/African American; AMR = Admixed American/Latino; ASJ = Ashkenazi Jewish; EAS = East Asian; FIN = Finnish; NFE = non-Finnish European; SAS = South Asian.

bioRxiv preprint doi: https://doi.org/10.1101/2023.03.19.533370; this version posted August 21, 2023. The copyright holder for this preprint (which was not certified by peer review) is the author/funder, who has granted bioRxiv a license to display the preprint in perpetuity. It is made available under aCC-BY 4.0 International license.

Figure S3

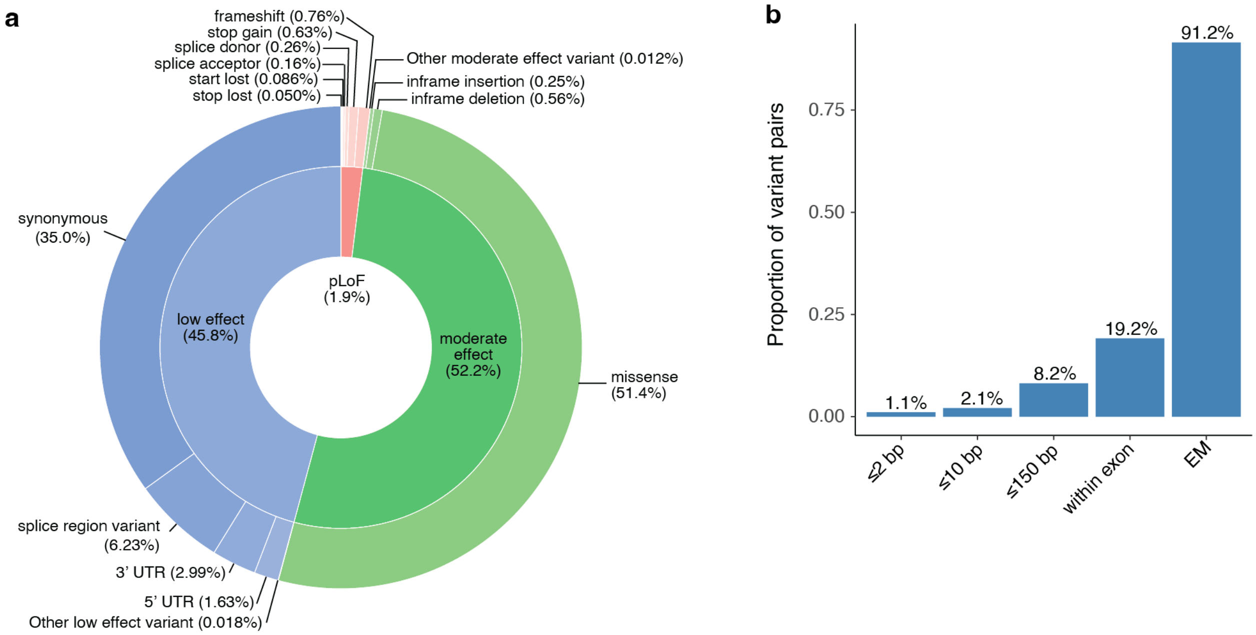

Supplementary Figure 3: a, Pie chart of variant effect annotations in the trio samples**.** Effect predictions are stratified among pLoF, moderate effect, and low effect variants. Percentages are shown in parentheses. **b,** Proportion of variant pairs falling within 2 bp, within 10 bp, within 150 bp, within the same exon, and proportion that can be phased using the EM algorithm and the gnomAD resource.

bioRxiv preprint doi: https://doi.org/10.1101/2023.03.19.533370; this version posted August 21, 2023. The copyright holder for this preprint (which was not certified by peer review) is the author/funder, who has granted bioRxiv a license to display the preprint in perpetuity. It is made available under aCC-BY 4.0 International license.

Figure S4

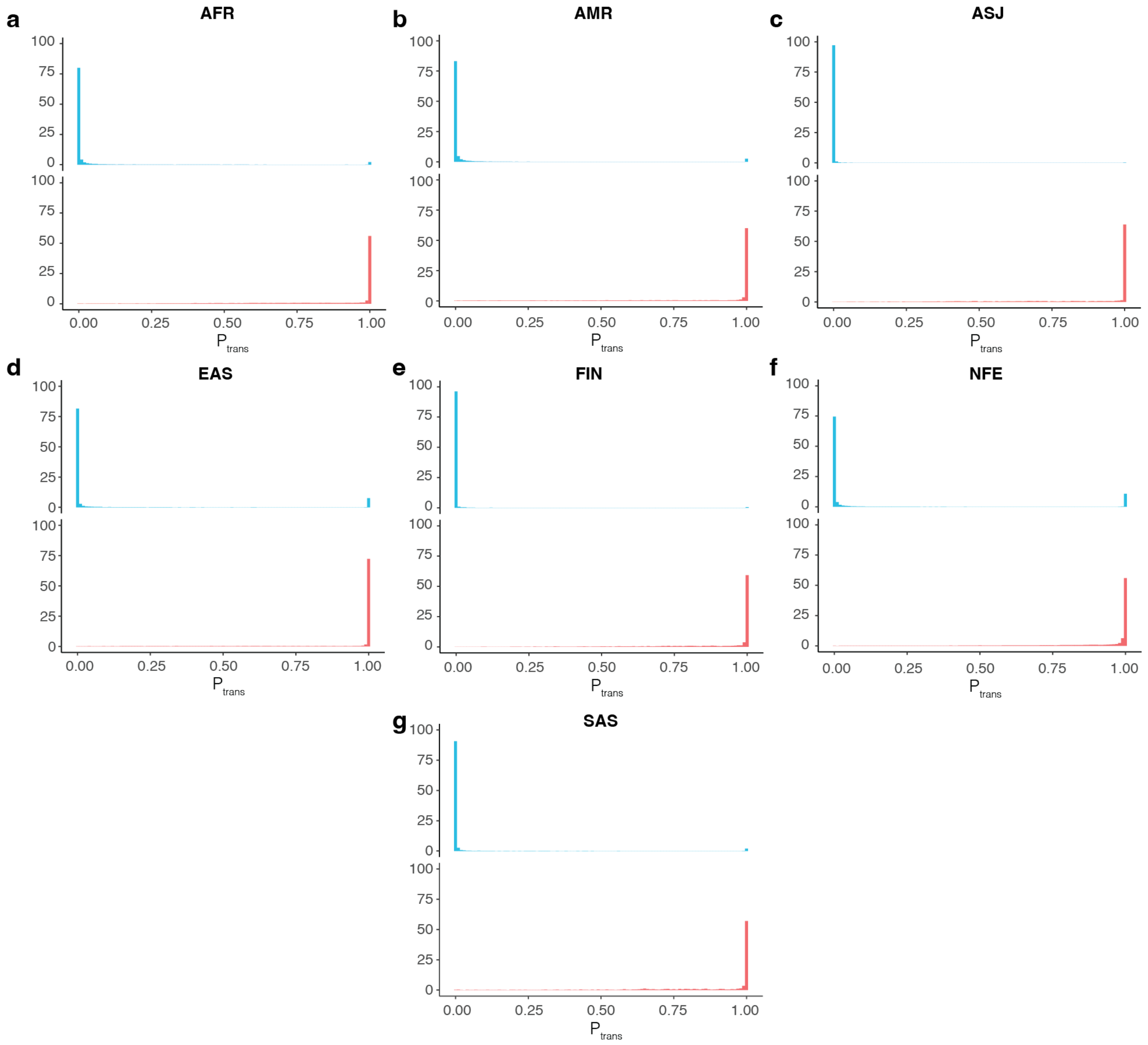

Supplementary Figure 4: a-g, Histogram of P_{trans} scores for variant pairs in *cis* (top, blue) and in *trans* (bottom, red) for each population. P_{trans} scores are population-specific. AFR = African/African American; AMR = Admixed American/Latino; ASJ = Ashkenazi Jewish; EAS = East Asian; FIN = Finnish; NFE = non-Finnish European; SAS = South Asian.

bioRxiv preprint doi: https://doi.org/10.1101/2023.03.19.533370; this version posted August 21, 2023. The copyright holder for this preprint (which was not certified by peer review) is the author/funder, who has granted bioRxiv a license to display the preprint in perpetuity. It is made available under aCC-BY 4.0 International license.

Figure S5

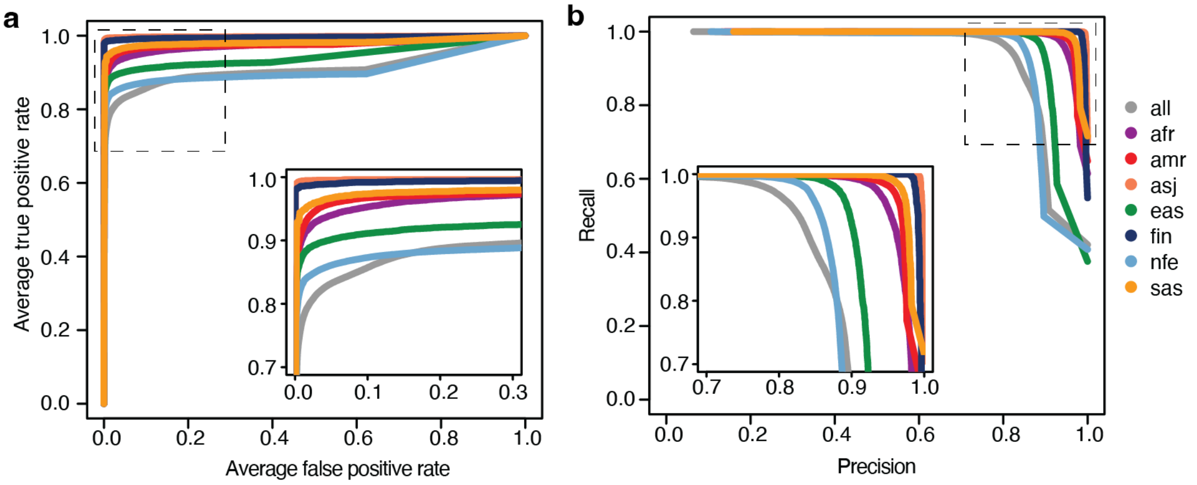

Supplementary Figure 5: Receiver-operator (**a**) and Precision-recall (**b**) curves for use of P_{trans} for distinguishing between variant pairs on same versus opposite haplotypes. Separate lines are shown for each genetic ancestry group. P_{trans} scores are population-specific. AFR = African/African American; AMR = Admixed American/Latino; ASJ = Ashkenazi Jewish; EAS = East Asian; FIN = Finnish; NFE = non-Finnish European; SAS = South Asian.

bioRxiv preprint doi: https://doi.org/10.1101/2023.03.19.533370; this version posted August 21, 2023. The copyright holder for this preprint (which was not certified by peer review) is the author/funder, who has granted bioRxiv a license to display the preprint in perpetuity. It is made available under aCC-BY 4.0 International license.

Figure S6

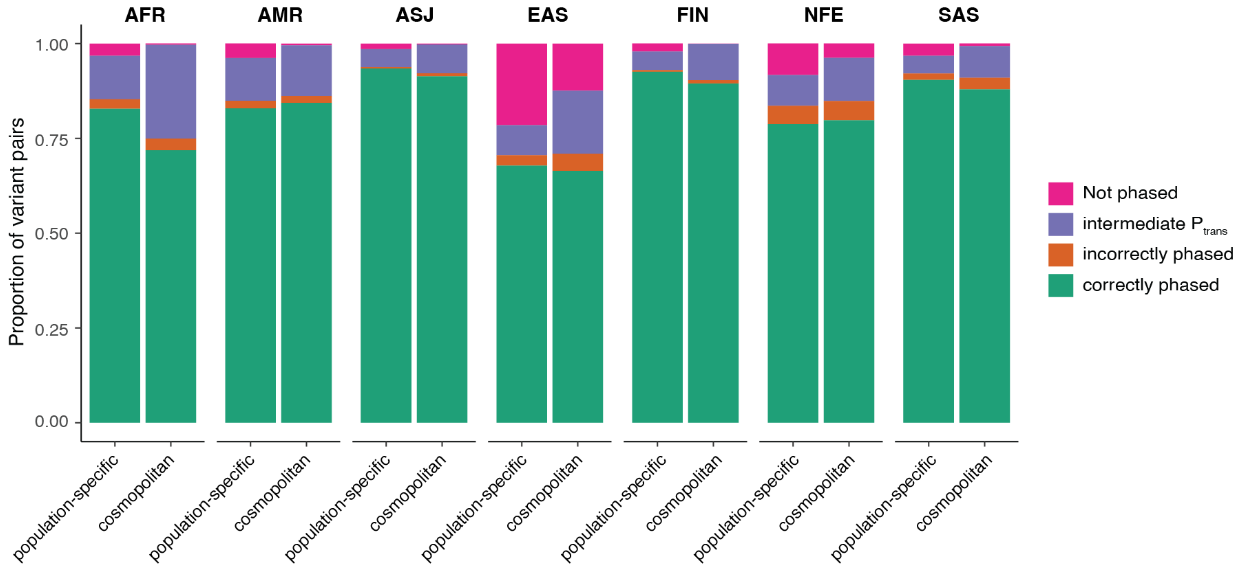

Supplementary Figure 6: Phasing performance for population-specific versus cosmopolitan P_{trans} scores for each population. AFR = African/African American; AMR = Admixed American/Latino; ASJ = Ashkenazi Jewish; EAS = East Asian; FIN = Finnish; NFE = non-Finnish European; SAS = South Asian.

bioRxiv preprint doi: https://doi.org/10.1101/2023.03.19.533370; this version posted August 21, 2023. The copyright holder for this preprint (which was not certified by peer review) is the author/funder, who has granted bioRxiv a license to display the preprint in perpetuity. It is made available under aCC-BY 4.0 International license.

Figure S7

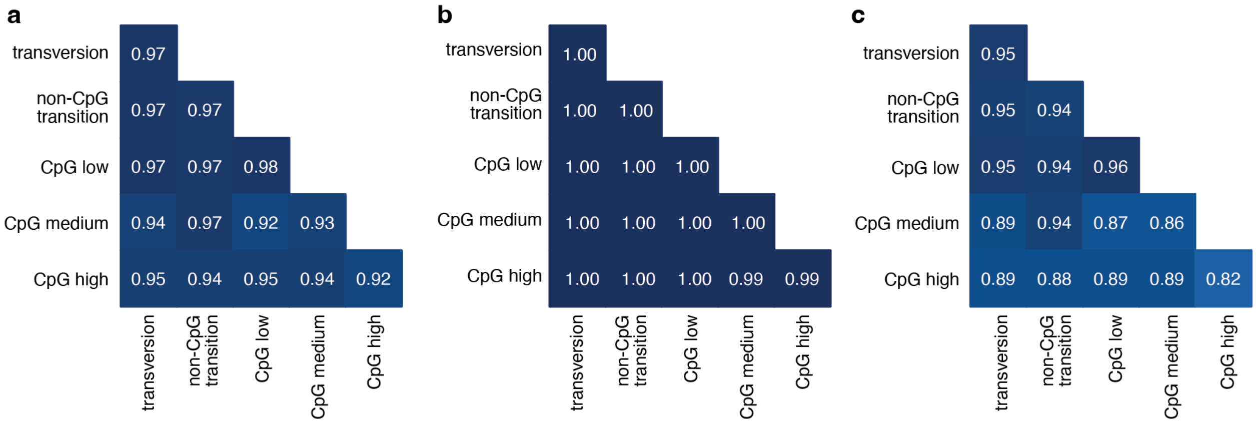

Supplementary Figure 7: Phasing accuracy for transversions, non-CpG transitions, and CpG transitions. CpG transitions are further stratified by degree of DNA methylation (low, medium, or high) as in Karczewski et al[20]. Shading of squares and numbers in each square represents phasing accuracy. Phasing accuracies are based on variant pairs seen in all populations and utilize population-specific P_{trans} estimates. Accuracy is shown for all variants **(a)**, variants in *trans* **(b),** and variants in *cis* **(c).**

bioRxiv preprint doi: https://doi.org/10.1101/2023.03.19.533370; this version posted August 21, 2023. The copyright holder for this preprint (which was not certified by peer review) is the author/funder, who has granted bioRxiv a license to display the preprint in perpetuity. It is made available under aCC-BY 4.0 International license.

Figure S8

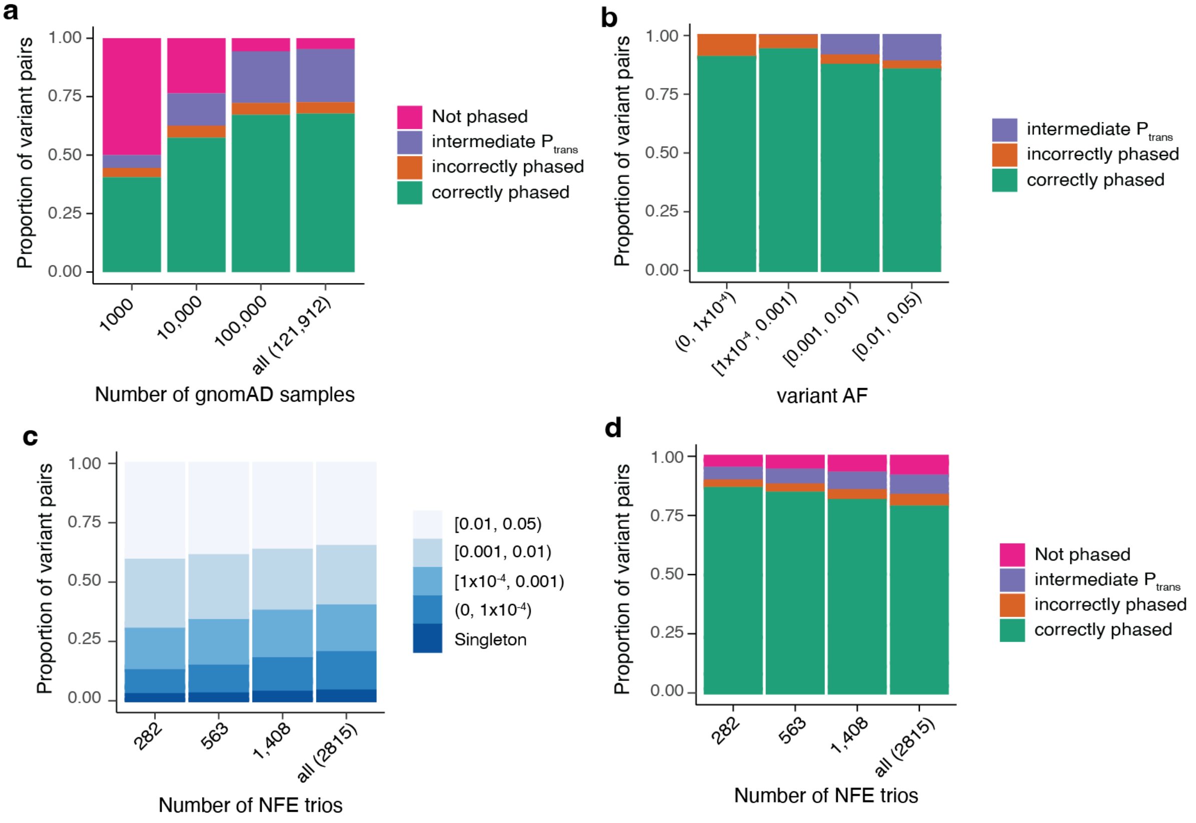

Supplementary Figure 8: a, Phasing performance when subsampling gnomAD to 1000, 10,000, 100,000 or using all samples. Phasing performance is based on cosmopolitan P_{trans} estimates and is calculated across trio samples from all populations. **b,** Phasing performance as a function of variant AF for the more common variant in a variant pair. Phasing performance is based on population-specific P_{trans} estimates and is calculated across trio samples from all populations. **c,** Proportion of variants falling into different AF bins when subsampling NFE gnomAD trios from 2815 trios down to 282, 563, or 1408 trios. Allele frequencies reflect the rarer variant in a variant pair. **d,** Phasing performance when subsampling NFE gnomAD samples as described in **c.**

bioRxiv preprint doi: https://doi.org/10.1101/2023.03.19.533370; this version posted August 21, 2023. The copyright holder for this preprint (which was not certified by peer review) is the author/funder, who has granted bioRxiv a license to display the preprint in perpetuity. It is made available under aCC-BY 4.0 International license.

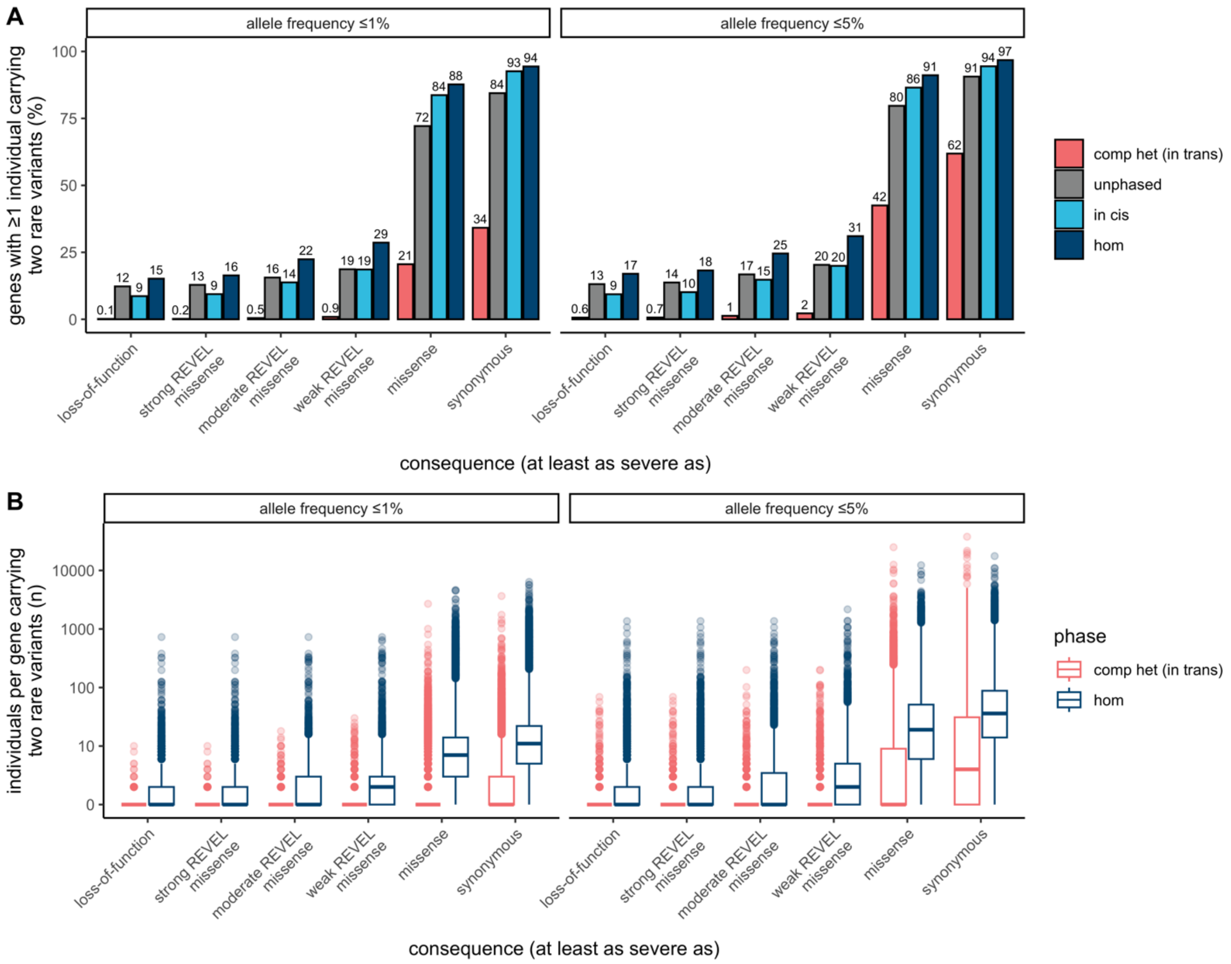

Supplementary Figure 9: a, Proportion of genes with one or more individuals in gnomAD carrying two rare variants at ≤ 1% and ≤ 5% AF stratified by predicted functional effect and phase. For compound heterozygous (comp het, in *trans*), unphased, and in *cis*, both variants in the variant pair must be annotated with a consequence at least as severe as the consequence displayed. **b,** Number of individuals per gene in gnomAD carrying two rare variants at ≤ 1% and ≤ 5% AF stratified by predicted functional effect and phase. For compound heterozygous (in *trans*) both variants in the variant pair must be annotated with a consequence at least as severe as the consequence displayed. In the box plots, the center line is the median, the box limits are the upper and lower quartiles, and the whiskers extend to the 1.5x the interquartile range. Any points shown are outliers. “comp het (in trans)” refers to compound heterozygous; “hom” refers to homozygous.
