# Supplementary material for: Inferring compound heterozygosity from large-scale exome sequencing data
Source: bioRxiv. 2023 Mar 23:2023.03.19.533370. Preprint. [Version 1] doi: 10.1101/2023.03.19.533370 (PMC10055215; doi:10.1101/2023.03.19.533370)
Supplement: Supplementary file 1 [file NIHPP2023.03.19.533370v1-supplement-3.pdf]

bioRxiv preprint doi: https://doi.org/10.1101/2023.03.19.533370; this version posted March 23, 2023. The copyright holder for this preprint (which was not certified by peer review) is the author/funder, who has granted bioRxiv a license to display the preprint in perpetuity. It is made available under aCC-BY 4.0 International license.

Supplementary Figures

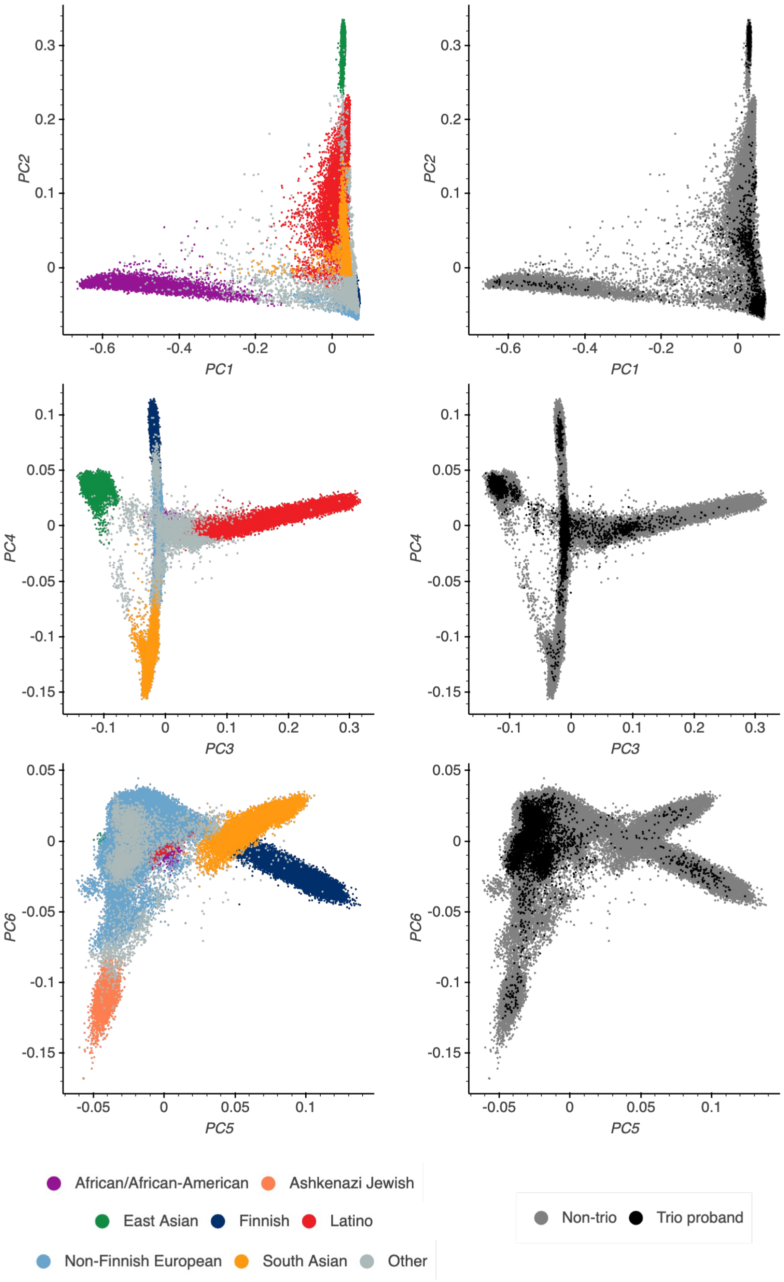

Figure S1: Principal component analysis (PCA) plot for the full gnomAD v2 cohort (left) and specifically for the trios (right, trios in black) included in this paper. The top row shows PC1 vs PC2, the middle row shows PC3 vs PC4, and the bottom row shows PC5 vs PC6. Genetic ancestry group labels for the global gnomAD populations were done as described in Karczewski et al. 2020[16].

bioRxiv preprint doi: https://doi.org/10.1101/2023.03.19.533370; this version posted March 23, 2023. The copyright holder for this preprint (which was not certified by peer review) is the author/funder, who has granted bioRxiv a license to display the preprint in perpetuity. It is made available under aCC-BY 4.0 International license.

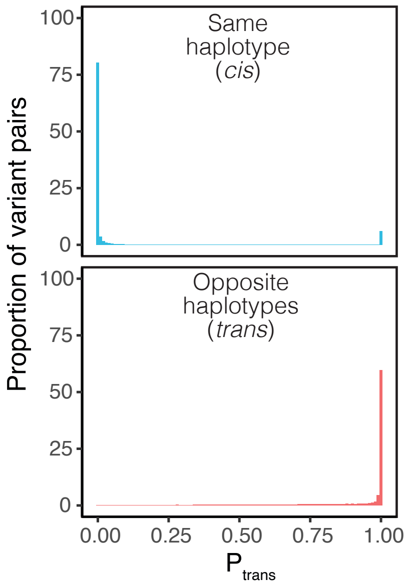

Figure S2: Histogram of P_{trans} values for variant pairs on the same haplotype (top) or opposite haplotypes (bottom). P_{trans} bin size of 0.01.

bioRxiv preprint doi: https://doi.org/10.1101/2023.03.19.533370; this version posted March 23, 2023. The copyright holder for this preprint (which was not certified by peer review) is the author/funder, who has granted bioRxiv a license to display the preprint in perpetuity. It is made available under aCC-BY 4.0 International license.

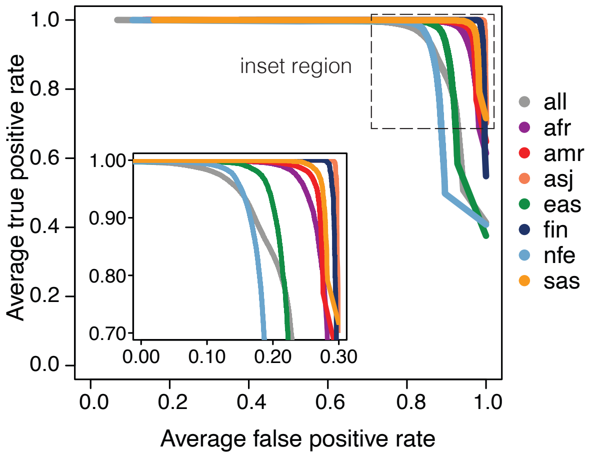

Figure S3: Precision-recall curves for use of P_{trans} for distinguishing between variant pairs on same versus opposite haplotypes. Separate lines are shown for each ancestry.

bioRxiv preprint doi: https://doi.org/10.1101/2023.03.19.533370; this version posted March 23, 2023. The copyright holder for this preprint (which was not certified by peer review) is the author/funder, who has granted bioRxiv a license to display the preprint in perpetuity. It is made available under aCC-BY 4.0 International license.

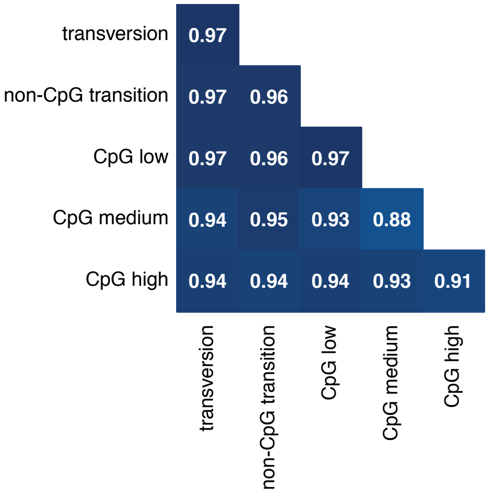

Figure S4: Phasing accuracy for transversions, non-CpG transitions, and CpG transitions. CpG transitions are further stratified by degree of DNA methylation (low, medium, or high) as in Karczewski et al 2020. Shading of squares and numbers in each square represent phasing accuracy. Phasing accuracies are based on variant pairs seen in all populations and utilize population-specific P_{trans} estimates.

bioRxiv preprint doi: https://doi.org/10.1101/2023.03.19.533370; this version posted March 23, 2023. The copyright holder for this preprint (which was not certified by peer review) is the author/funder, who has granted bioRxiv a license to display the preprint in perpetuity. It is made available under aCC-BY 4.0 International license.

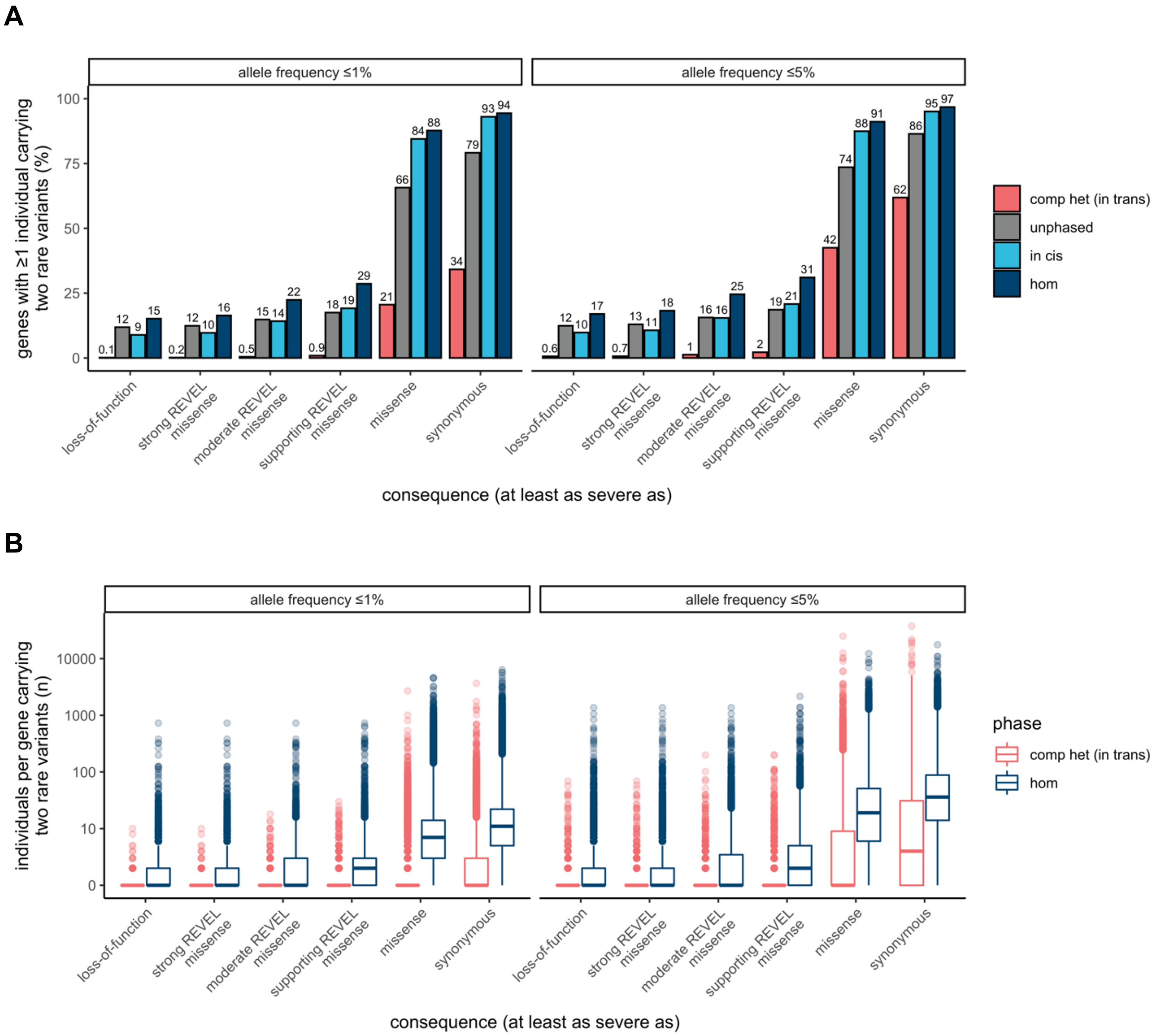

Figure S5: A) Proportion of genes with one or more individuals in gnomAD carrying two rare variants at ≤1% and ≤5% allele frequency stratified by predicted functional effect and phase. For comp het (in *trans*), unphased, and in *cis*, both variants in the variant pair must be annotated with a consequence at least as severe as the consequence displayed. **B)** Number of individuals per gene in gnomAD carrying two rare variants at ≤1% and ≤5% allele frequency stratified by predicted functional effect and phase. For compound heterozygous (in *trans*) both variants in the variant pair must be annotated with a consequence at least as severe as the consequence displayed. comp het, compound heterozygous; hom, homozygous.

bioRxiv preprint doi: https://doi.org/10.1101/2023.03.19.533370; this version posted March 23, 2023. The copyright holder for this preprint (which was not certified by peer review) is the author/funder, who has granted bioRxiv a license to display the preprint in perpetuity. It is made available under aCC-BY 4.0 International license.

Supplemental Tables

Table S1. CMG diagnostic variants. In this table, we provide details about the presumed bi-allelic causal variants from 293 individuals from the Broad Institute Center for Mendelian Genetics. For each variant pair, we provide the gene symbol ("gene_name"), information about the position and alleles of both variants, whether both of the variants were singletons in gnomAD ("singleton_singleton") and seen in the same individual or not, the estimated P_{trans} value, the predicted phase based on the P_{trans} value ("phase_prediction"), and the known phase from phase by transmission when available.

Table S2. Manual curation results for compound heterozygous loss-of-function variants. Here, we provide the variant curation information for the 28 genes that have predicted compound heterozygous loss-of-function variants with allele frequency ≤ 1%. For every predicted compound heterozygous variant pair, we provide the gene symbol, maximum allele frequency in the gnomAD exomes from the two variants ("variant_pair_max_af"), the number of individuals who carry the variant pair ("n_individuals"), information about the position and alleles of both variants, any manual curation flags e.g., mapping error for the variants, and the final loss-of-function curation for both variants as well as the variant pair ("high_confidence_human_knock_out").
